# Evidence of transmission and dissemination of diverse *bla*_NDM-5_-producing *Escherichia coli* clones between refugee and host communities and their environment: a multicenter cross-sectional study

**DOI:** 10.1128/aem.01625-25

**Published:** 2025-11-04

**Authors:** Souad Fayad, Dina Daaboul, Issmat I. Kassem, Aula Abbara, Casey L. Cazer, Kathryn J. Fiorella, Kevin J. Cummings, Iman Yassine, Monzer Hamze, Khaled El Omari, Fouad Dabboussi, Saoussen Oueslati, Thierry Naas, Marwan Osman

**Affiliations:** 1 Laboratoire Microbiologie Santé et Environnement (LMSE), Doctoral School of Sciences and Technology, Faculty of Public Health, Lebanese University, Tripoli, Lebanon; 2 Team 'Resist' UMR1184, 'Immunology of Viral, Auto-Immune, Hematological and Bacterial Diseases, IMVA-HB', INSERM, Université Paris-Saclay, CEA, LabEx LERMIT, Faculty of Medicine, Le Kremlin-Bicêtre, France; 3 Department of Biology, Faculty of Arts and Sciences, Holy Spirit University of Kaslik, Jounieh, Lebanon; 4 Center for Food Safety, Department of Food Science and Technology, University of Georgia 92569, Griffin, Georgia, USA; 5 Faculty of Agricultural and Food Sciences, American University of Beirut 11238, Beirut, Lebanon; 6 Syria Public Health Network, London, United Kingdom; 7 Department of Infectious Diseases, Imperial College of London, St Mary's Hospital 4615, London, United Kingdom; 8 Department of Public and Ecosystem Health, College of Veterinary Medicine, Cornell University 5922, Ithaca, New York, USA; 9 Nuffield Department of Population Health, University of Oxford 6396, Oxford, United Kingdom; 10 Microbiology Department, Clinical Laboratory, Nini Hospital 237177, Tripoli, Lebanon; 11 Quality Control Center Laboratories at the Chamber of Commerce, Industry & Agriculture of Tripoli & North Lebanon, Tripoli, Lebanon; 12 Department of Neurosurgery, Yale University School of Medicine 537605, New Haven, Connecticut, USA; 13 Yale Institute for Global Health 198927 , New Haven, Connecticut, USA; 14 Cornell Atkinson Center for Sustainability, Cornell University 5922, Ithaca, New York, USA; Centers for Disease Control and Prevention, Atlanta, Georgia, USA

**Keywords:** public health, refugee, humanitarian crisis, conflict, one health, global health, NDM-5, carbapenemase, antimicrobial resistance, *Escherichia coli*

## Abstract

The global spread of carbapenemase-producing *Escherichia coli* (CP-Ec) poses a significant public health threat, with particularly severe consequences for vulnerable populations in resource-limited settings. To address this, we conducted in-depth genetic analyses and examined the relatedness of CP-Ec isolates recovered from hospitalized patients, refugees, animals, water, and environmental sources within refugee camps and in marginalized host communities in Lebanon. Nineteen putative CP-Ec isolates, identified by MALDI-TOF MS and designated as community isolates, harbored either NDM (*n* = 17) or OXA-48-like (*n* = 2) carbapenemases. We used whole-genome sequencing (WGS) to characterize the resistomes and sequence types of these isolates. To further examine genetic relationships and transmission dynamics, we also analyzed publicly available (EnteroBase) CP-Ec genomes from Lebanon (*n* = 64) and across the globe (*n* = 447 recovered in 2022) alongside 31 additional clinical CP-Ec isolates from the same geographic region. The community isolates belonged to ST10, ST167, ST361, ST410, ST617, ST648, ST940, ST1284, and ST5842. Both community and clinical CP-Ec isolates carried multiple acquired antimicrobial resistance (AMR) genes and chromosomal mutations, with 82% harboring the *bla*_NDM-5_ gene. Core-genome SNP analysis showed that refugee isolates clustered with global CP-Ec genomes, highlighting their genomic relatedness and potential for geographical dissemination. Furthermore, integration of our data with previously reported Lebanese genomes demonstrated the spread of *bla*_NDM-5_-carrying *E. coli* across different hosts and niches, emphasizing the complex interplay of AMR within the human-animal-environment interface. The coexistence of carbapenemase genes with mobile genetic elements that enable horizontal gene transfer raises concerns about the emergence of highly resistant and hypervirulent CP-Ec lineages, especially in vulnerable populations and settings.

**IMPORTANCE:**

The global rise of CP-Ec strains harboring *bla*_NDM-5_ has been increasingly documented in clinical settings. However, little is known about their emergence and transmission in refugee settlements. This study provides a high-resolution genomic characterization of CP-Ec isolated from human, animal, water, and environmental sources in refugee settlements and surrounding host communities. By integrating whole-genome sequencing data from clinical isolates collected in Lebanese hospitals, we reveal genetically related strains in both community and healthcare settings, highlighting the potential introduction of community-acquired strains into clinical environments and vice versa. The widespread detection of *bla*_NDM-5_ across multiple reservoirs suggests sustained circulation beyond hospital settings. The identification of CP-Ec in river water used for irrigation and emptying into the Mediterranean Sea highlights wider environmental dimensions that may drive regional dissemination of AMR. Our findings highlight the urgent need for One Health-based AMR surveillance strategies to track the spread of carbapenem-resistant pathogens in high-risk settings.

## INTRODUCTION

Antimicrobial resistance (AMR) is one of the most pressing global health challenges of the 21^st^ century, with significant implications for both human and animal health and socioeconomic stability. The widespread and often indiscriminate use of antimicrobials in healthcare, agriculture, and animal husbandry has accelerated the emergence of multidrug-resistant (MDR) pathogens, including those resistant to last-resort antimicrobials such as carbapenems and novel β-lactam-β-lactamase inhibitor combinations. Without urgent interventions, AMR is projected to directly cause 1.91 million direct deaths and contribute to 8.22 million associated deaths annually by 2050. Over the 25-year period from 2025 to 2050, this equates to a staggering 39.1 million direct deaths and 169 million associated fatalities worldwide (1).

AMR also threatens to destabilize healthcare systems, deepen poverty, and exacerbate health inequities, particularly in low- and middle-income countries (LMICs). Refugee populations and their marginalized host communities in LMICs are highly vulnerable to the AMR crisis and serve as critical hotspots for AMR emergence, spread, and/or amplification (2). Overcrowded settlements, inadequate sanitation, and fragmented healthcare services create conditions that promote the spread of MDR infections in both refugee and host communities. Limited access to quality healthcare often spurs the inappropriate use of antimicrobials, whereas the lack of diagnostic capacity and effective surveillance systems allows MDR pathogens to circulate undetected. In these settings, AMR not only threatens individual health outcomes but also poses a broader public health risk, as resistant strains spread across borders and undermine global efforts to control infectious diseases (3).

Lebanon, a country with the highest refugee-to-population ratio in the world, exemplifies this crisis. Over 1.5 million displaced Syrian refugees primarily reside in two of the most marginalized areas in the country, namely North Lebanon and the Bekaa Valley in the East of Lebanon. Refugees in the areas live under harsh conditions akin to other disenfranchised groups, including Palestinian and Iraqi refugees and impoverished Lebanese communities (4). Syrian refugees and low-income Lebanese alike grapple with extreme financial hardship, personal insecurity, and the aftermath of protracted compounding crises, such as the Lebanese economic collapse, the COVID-19 pandemic, armed conflicts, and political unrest (4). These converging factors have further eroded Lebanon’s already fragile healthcare system, leaving both refugee and host populations with limited access to essential medical services and promoting inappropriate antimicrobial use in community settings (5). This bidirectional vulnerability creates a dangerous cycle, where MDR pathogens circulate between refugee and host populations, amplifying the public health threat (6).

Equally concerning is the role of veterinary and environmental factors in the spread of AMR, particularly in marginalized communities where AMR trends are exacerbated by eroded infrastructure and environmental barriers. The exchange of MDR pathogens and AMR determinants across the human-animal-environment continuum is a key driver of resistance, contaminating various ecological niches and creating persistent reservoirs of MDR bacteria. Taken together, this situation can potentially become a vicious cycle of spreading and acquiring evolving MDR pathogens.

Despite growing anecdotal evidence of carbapenem resistance, particularly the emergence of *bla*_NDM-5_-producing Enterobacterales in clinical settings in Lebanon (7), there remains a significant gap in data on carbapenem resistance in refugees and associated settings. To address this critical knowledge gap, we conducted an in-depth characterization of carbapenemase-producing *Escherichia coli* (CP-Ec) isolated from vulnerable Syrian refugees, domesticated animals, water sources, and the environment within the refugee camps, as well as from hospitalized Lebanese individuals in the refugee-hosting community. This study aimed to investigate the genetic relatedness and transmission dynamics of *bla*_NDM-5_-harboring *E. coli* at the human-animal-environment interface, focusing on the interactions between individuals and families within the refugee camps and between the camps and the surrounding host communities.

## MATERIALS AND METHODS

### Study design

This is a multicenter cross-sectional community-based study, which was carried out from July to August 2022 across four refugee camps of different sizes—Al-Qlayaat (Camp 1, 160 tents), Al-Mida (Camp 2, 53 tents), Sammouniyeh (Camp 3, 45 tents), and Mhamra (Camp 4, 220 tents)—located in Akkar, a rural governorate in Northern Lebanon. These camps collectively house approximately 10,000 Syrian refugees. We randomly selected three to six families per camp, ensuring representation from different camp areas by including tents located both at the edges and in the center of each camp. Tents were first assigned numbers according to their location, and then, a random number generator was used separately in each camp to select the families for inclusion. Additionally, we included six families from neighboring communities who voluntarily participated. Despite economic challenges, Akkar hosts the largest number of refugees in Lebanon, with ~300,000 Lebanese residents sheltering over 150,000 Syrians, creating an almost 1:2 ratio between the refugee and host populations (8). To achieve the study objectives with a 5% margin of error and a 95% confidence level, the minimum required sample size was calculated to be 59 using the Raosoft sample size calculator (http://www.raosoft.com/samplesize.html). This calculation was based on a population size of 450,000 and an estimated 4% prevalence of CP-Ec carriage in community settings in Lebanon (9). A total of 213 community samples were collected from four refugee camps (*n* = 178) and neighboring host communities (*n* = 35) and analyzed in this study (Table S1). The samples included human (*n* = 95) and animal (*n* = 8) stool, river water (*n* = 2), drinking water (*n* = 55), and environmental swabs (*n* = 53) (Table S2). Upon enrolling a family, we obtained stool samples from its members, along with their household drinking water and environmental swabs from key contact surfaces, such as faucets and floors. Drinking water originated from multiple sources, including wells within and outside the camps and private vendors. The water was typically collected in central tanks within the camps and then distributed to tents, where it was stored in various small containers (e.g., plastic water bottles). River water samples were obtained from camps 1 and 2, where rivers run adjacent to the refugee settlements. Animal samples were collected exclusively from camp 2, which was the only camp where residents raised livestock near their tents.

### Inclusion criteria for human participants

Inclusion criteria for all study participants were as follows: (i) participants had to be residents of the refugee camps or neighboring communities within the main county/district hosting these camps, and (ii) the participants were 1 month or older, had no symptoms of infection, and had not taken antimicrobials in the last 6 months.

### Collection of demographic data and biological samples

Enrollment was conducted at the family level. Among those approached, only a subset agreed to participate. Once a family consented to participate, each member was then individually invited to confirm their participation and provide informed consent. Participants were provided with comprehensive information regarding the study’s objectives, methodology, and sample collection procedures. Following this, written informed consent was obtained from each participant or their legal representative. Data were collected through face-to-face interviews using a structured questionnaire, with guardians responding on behalf of young children. Participants self-collected stool samples. For children still wearing diapers, samples were obtained directly from the disposable diapers, whereas for older children, collection was assisted by their guardian. Trained investigators collected animal rectal swabs as well as water and environmental fomite samples from camps and households. All samples were subsequently transported to the laboratory in a cooler (2°C–10°C) and processed within 6 h of collection.

### Bacterial isolation and identification

Water testing was conducted by aseptically collecting approximately 500 mL of drinking water from family-used bottles. A 100 mL aliquot of water was then filtered through 0.22 µm Millipore membranes (Sigma-Aldrich, St. Louis, MO). CP-Ec isolation from human and animal rectal swab samples, water membranes, and environmental swabs was performed by culturing on RAPID'*E. coli* 2 agar plates (BioRad, Hercules, CA) supplemented with ertapenem (2 µg/mL) (Sigma-Aldrich, St Louis, MO, USA) as previously described (9). Suspected colony-forming units (CFUs) of *E. coli* (violet to pink colonies) were selected, purified, and subsequently confirmed using matrix-assisted laser desorption/ionization-time of flight (MALDI-TOF) mass spectrometry (MS) (Vitek MS, bioMérieux, Marcy L'Etoile, France). Notably, when multiple colonies of *E. coli* were retrieved from a sample, we randomly selected one colony for further analysis.

### Collection of clinical CP-Ec isolates

To assess the genetic relationships and transmission dynamics and compare the resistance patterns of CP-Ec isolates found in the refugee camps and neighboring communities, 31 CP-Ec isolates belonging to the same sequence types (STs) associated with refugees were retrospectively retrieved from clinical samples of hospitalized patients, either infected (*n* = 14) with or colonized (*n* = 17) by these bacteria. These isolates were collected between 2021 and 2022 by the bacteriology laboratories of two major tertiary care hospitals within the same geographic region, namely the Nini Hospital in Tripoli and El Youssef Hospital Center in Akkar. As part of routine clinical testing and in accordance with internal hospital policies, the isolates were stored in the hospital’s strain banks based on their AMR susceptibility profiles (Table S3).

### Antimicrobial susceptibility testing

The isolates were confirmed for ertapenem resistance using the Kirby-Bauer disk diffusion assay. Carbapenemase activity in both community and hospital ertapenem-resistant isolates was then confirmed using the Carba NP test and the NG-TEST CARBA-5 lateral flow immunoassay (NG-Biotech, Guipry, France), enabling phenotypic identification of the five most prevalent carbapenemase families: NDM, IMP, VIM, OXA-48, and KPC. Antimicrobial susceptibility profiles and minimum inhibitory concentrations (MICs) were determined using the broth microdilution method (Sensititre, ThermoFisher, Grenoble, France) to assess both classic and novel β-lactam and non-β-lactam antimicrobials of clinical and veterinary relevance, including mecillinam, cefoxitin, cefotaxime, ceftazidime, ceftazidime-avibactam, cefalexin, aztreonam, aztreonam-avibactam, cefepime, ceftolozane-tazobactam, cefiderocol, ertapenem, imipenem, imipenem-relebactam, meropenem, meropenem-vaborbactam, norfloxacin, levofloxacin, ciprofloxacin, delafloxacin, amikacin, gentamicin, tobramycin, streptomycin, apramycin, neomycin, tetracycline, tigecycline, eravacycline, chloramphenicol, florfenicol, colistin, fosfomycin, nitrofurantoin, trimethoprim, sulfamethoxazole, and trimethoprim-sulfadiazine. The MIC testing for cefiderocol was performed using UMIC Cefiderocol strip testing as previously described (10). The Kirby-Bauer disk diffusion assay was used to determine susceptibility to antimicrobials that were not assessed by the broth microdilution method, including amoxicillin, amoxicillin-clavulanate, ticarcillin, temocillin, piperacillin, ticarcillin-clavulanate, piperacillin-tazobactam, moxalactam, kanamycin, and netilmicin. Susceptibility patterns were interpreted according to the Clinical and Laboratory Standards Institute (CLSI-M100 2024) guidelines, where applicable. For aztreonam-avibactam, interpretations were based solely on aztreonam breakpoints, and for ceftiofur, apramycin, neomycin, and streptomycin, veterinary-specific breakpoints were used (https://www.sfm-microbiologie.org/boutique/casfm-vet-2023/). The *E. coli* ATCC 7624 and *E. coli* ATCC 25922 strains were used for quality control.

### Whole-genome sequencing and bioinformatic analyses

Total DNA was extracted from community and hospital CP-Ec isolates using the PureLink Genomic DNA Mini-Kit (ThermoFisher Scientific, Waltham, MA, USA) according to the manufacturer’s protocol and stored at −20°C. The extracted genomic DNA was then used for library preparation with the NEBNext Ultra II FS DNA Library Prep Kit for Illumina (NEB, France), following the manufacturer’s instructions. Whole-genome sequencing was conducted on an Illumina NextSeq 500 platform. After sequencing, raw reads were *de novo* assembled using the CLC Genomics 10.2 software (Qiagen, Les Ulis, France), and CP-Ec genomes were analyzed online using software available at the Center for Genomic Epidemiology-CGE (https://cge.food.dtu.dk/). The latter included multilocus sequence typing (MLST) with the CGE MLST 2.0.9 software to determine STs. AMR genes were identified through ResFinder (v4.6.0) using default cutoffs (90% minimum identity and 60% minimum coverage). Pathogenicity and virulence genes were identified through PathogenFinder (V2.0.5) and VirulenceFinder (V2.0) using 100% minimum identity and 100% minimum coverage, respectively. Plasmid replicon types were detected using PlasmidFinder (v2.1) with the default thresholds of 95% minimum identity and 60% minimum coverage. The generated raw reads were uploaded to the EnteroBase web resource. Trimming, *de novo* assembly, quality control, and annotation were performed through the associated EnteroBase pipeline (http://enterobase.warwick.ac.uk/). The PBP3-encoding gene (*ftsI*), *ompC,* and *omp*F sequences were extracted and aligned with the wild-type *E. coli* reference strain (acc no. U00096.3) using the BLAST tool from the National Center for Biotechnology Information (NCBI). Parsnp (v1.7.4) (http://harvest.readthedocs.io/en/latest/content/parsnp.html) was used to align the core genomes of CP-Ec strains from the present study (*n* = 50) against the reference genomes, including ST410 (PRJNA869915), ST648 (PRJNA654992), ST617 (PRJNA224116), ST167 (PRJNA298278), and ST361 (PRJNA836696). To investigate genetic relationships and transmission dynamics, we included genomes of strains belonging to the same STs - ST167, ST361, ST410, ST617, and ST648, regardless of their resistance phenotype. These genomes, previously isolated in Lebanon and available in EnteroBase (Table S4), were analyzed alongside the community and clinical strains from our study (11). A maximum-likelihood phylogenetic tree was then generated within EnteroBase using SNP Project. The final phylogenetic tree was visualized and graphically edited using iTOL v5.6 (12) and was midpoint rooted. Afterward, the matrix of SNP distances was identified on the core gene alignment, generating a core SNP alignment. The distance between two *E. coli* isolates from different sources, lower than the ≤10 SNP threshold, was evaluated as clonal spread (13). To investigate whether the CP-Ec isolates from refugees were phylogenetically related to globally circulating strains, we constructed a core genome MLST (cgMLST) phylogenetic tree of CP-Ec using 447 publicly available genomes in EnteroBase, including CP-Ec belonging to ST10 (*n* = 14), ST167 (*n* = 186), ST361 (*n* = 87), ST410 (*n* = 90), ST617 (*n* = 29), ST648 (*n* = 28), ST940 (*n* = 4), and ST1284 (*n* = 9) isolated in 2022 (Table S5).

### Statistical analysis

Data from the questionnaire and laboratory analyses were analyzed using R software (R Core Team, version 4.3.1) via RStudio (version 2025.05.0 + 496). Fisher’s exact test was used to assess differences in the prevalence of AMR genes between community- and hospital-derived CP-Ec isolates. Statistical tests were two-sided, with a type I error set at α = 0.05.

## RESULTS

### Sample collection and detection of CP-Ec isolates

Ninety-five individuals (37 males and 58 females) from families living in camps were enrolled. In each camp, three to six families participated, with the number of individuals per camp ranging from 11 to 23. Additionally, six families (five Lebanese and one Syrian) from outside the camps but within the same geographic area were included. The median age of participants was 16 years (range: 2 months to 66 years). CP-Ec was detected in 10 individuals (10.5%) from seven families, including eight Syrian refugees in Camps 2, 3, and 4, and two individuals (one Syrian refugee and one Lebanese) from the host community. Nine environmental samples also carried CP-Ec, including animal feces (2/8; 25%), water sources (3/57; 5.3%), and environmental swabs (4/53; 7.5%).

In parallel, clinical CP-Ec isolates collected between 2021 and 2022 from hospitalized patients (*n* = 31; 19 males and 12 females) in the same geographic region were analyzed (Table S3). The median patient age was 68 years (range: 1 month to 86 years). Most of these strains (17/31; 54%) were obtained from rectal and axillary swab screenings of patients with risk factors. The remaining 46% (14/31) were considered infection-related because they were isolated from peritoneal fluid, sputum, and urine samples. During the 2-year period, the number of infection-related isolates increased more than the number of screening isolates. A nearly 1.2-fold increase in the proportion of infection-related isolates (from 42.1% in 2021 to 50% in 2022) was observed, whereas the proportion of screening isolates decreased from 57.9% to 50% over the same period.

### Carbapenemase detection and susceptibility testing of CP-Ec isolates

**Fig 1:**
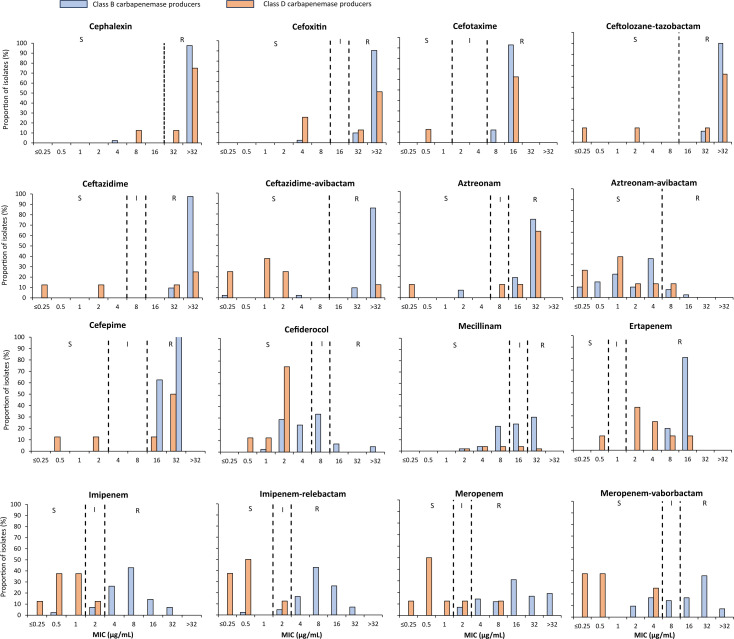
β-lactam MIC distributions of community (*n* = 19) and clinical (*n* = 31) carbapenemase-producing *Escherichia coli* (CP-Ec) isolates recovered in this study.

**Fig 2:**
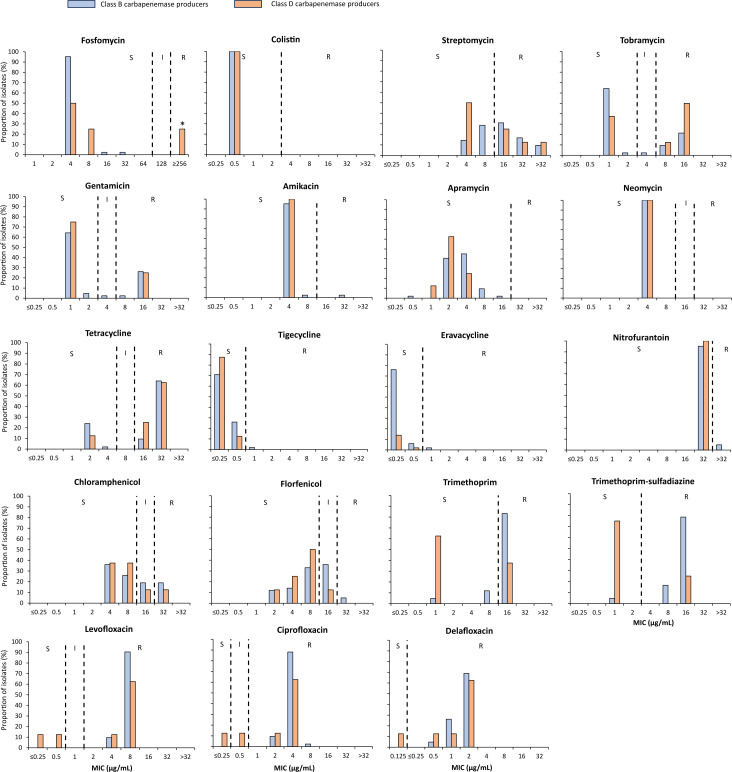
Non-β-lactam MIC distributions of community (*n* = 19) and clinical (*n* = 31) carbapenemase-producing *Escherichia coli* (CP-Ec) isolates recovered in this study. *Our broth microdilution method does not allow the determination of the exact MIC to fosfomycin when it exceeds 32 µg/mL. Therefore, isolates with MIC >32 µg/mL were designated as MIC ≥256 µg/mL, as resistance was confirmed using the disk diffusion method.

The isolates exhibited various levels of resistance to ertapenem (MIC = 0.5–16 μg/mL), with positive Carba NP and NG-TEST CARBA-5 test results. NDM (*n*  = 42) and OXA-48-like (*n*  = 8) were the only carbapenemases detected in these isolates. Different AMR phenotypes were observed (Table S6). Using disk diffusion and broth microdilution methods, over 90% of CP-Ec isolates were found to be resistant to nearly all antimicrobials tested routinely in Lebanon, including amoxicillin, amoxicillin-clavulanate, ticarcillin, ticarcillin-clavulanate, temocillin, piperacillin, piperacillin-tazobactam, cephalexin, cefoxitin, cefotaxime, ceftazidime, ceftolozane-tazobactam, aztreonam, ertapenem, ciprofloxacin, levofloxacin, and delafloxacin. High rates of resistance were also observed against novel β-lactam agents, including ceftolozane-tazobactam (96%; *n* = 48/50), imipenem-relebactam (84%), ceftazidime-avibactam (82%), meropenem-vaborbactam (74%), and cefiderocol (38%). Notably, aztreonam-avibactam was the most effective β-lactam, with 90% of isolates remaining susceptible (Fig. 1). Furthermore, CP-Ec isolates remained consistently susceptible to colistin (100%, MIC_90_ ≤0.5 µg/mL), apramycin (100%, MIC_90_ ≤16 µg/mL), neomycin (100%, MIC_90_ ≤4 µg/mL), amikacin (94%, MIC_90_ ≤8 µg/mL), tigecycline (98%, MIC_90_ ≤0.5 µg/mL), eravacycline (98%, MIC_90_ ≤0.5 µg/mL), nitrofurantoin (96%, MIC_90_ ≤32 µg/mL), and fosfomycin (96%, MIC_90_ ≤32 µg/mL) (Fig. 2). No significant differences in resistance patterns were observed between community- and hospital-associated isolates (Table S7).

### Molecular determinants of antimicrobial resistance

**Fig 3:**
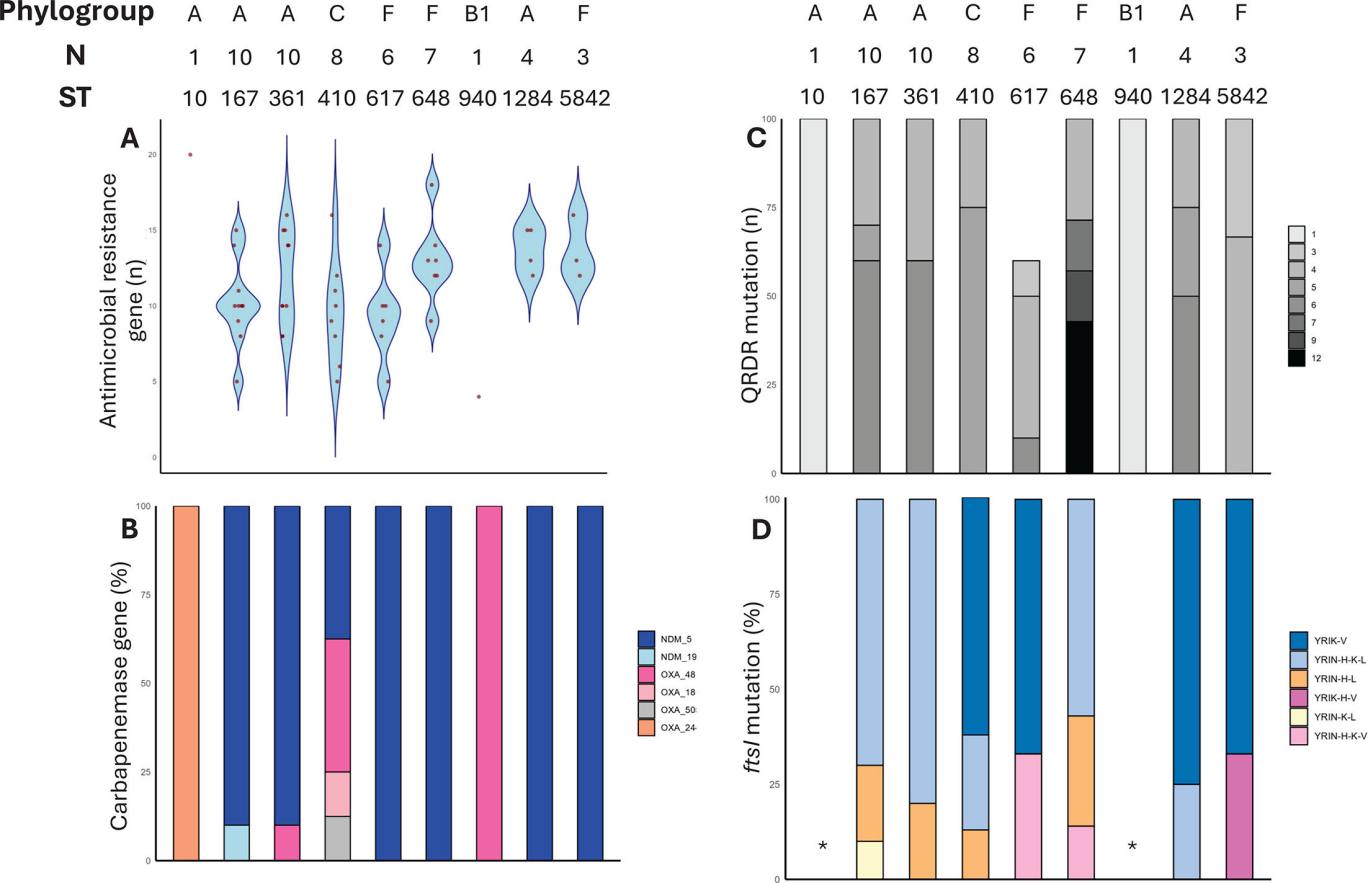
Distribution of antimicrobial resistance (AMR) determinants among community (*n* = 19) and clinical (*n* = 31) carbapenemase-producing *Escherichia coli* (CP-Ec) isolates recovered in this study. (**A**) Number of acquired AMR genes identified in each sequence type (ST). (**B**) Proportion of isolates stratified by carbapenemase genes across STs. (**C**) Proportion of isolates according to the number of mutations in QRDR. (**D**) Proportion of isolates harboring insertions in the *ftsI* gene encoding PBP3, including associated amino acid substitutions. The YRIN-H-L insertion is associated with Q227H and I532L; YRIN-H-K-L with Q227H, E349K, and I532L; YRIN-K-L with E349K and I532L; YRIN-H-K-V with Q227H, E349K, and A413V; YRIK-V with A413V; and YRIK-H-V with Q227H. '*' denotes absence of *ftsI* mutations. Violin plot was not applied in panel A for STs represented by a single strain.

The whole-genome sequencing analysis of the 50 CP-Ec isolates identified a total of 53 AMR genes (Table S8), conferring resistance to clinically- and veterinary-relevant antimicrobial classes, including last-resort agents. The most prevalent carbapenemase gene was *bla*_NDM-5_ (82%; 41/50), followed by *bla*_OXA-48_ (10%), *bla*_OXA-244_ (2%), *bla*_OXA-181_ (2%), *bla*_OXA-505_ (2%), and *bla*_NDM-19_ (2%). Stratification by source showed that *bla*_NDM-5_ was more frequent among community-acquired isolates (17/19, 89.5%) compared with hospital-acquired CP-Ec (24/31, 77.4%; OR = 2.48, 95% CI = 0.55–12.8, *P* = 0.45). The two remaining community isolates carried *bla*_OXA-48_ and *bla*_OXA-244_, respectively. Among the remaining hospital isolates, one harbored *bla*_NDM-19_, four carried *bla*_OXA-48_, one carried *bla*_OXA-505_, and another carried *bla*_OXA-181_. Resistance to ertapenem was observed in all isolates except the *bla*_OXA-244_-producer (O102C4), which remained susceptible (Table S6). Notably, 96% of the isolates harbored mutations in *ftsI* (YRIN and YRIK insertions), leading to structural modifications in PBP3 (Fig. 3). Additional mutations in the *ompC* and *ompF* porin genes were also observed among isolates with *ftsI* mutations, potentially compounding their resistance phenotype. Of note, the ST617 and ST1284 CP-Ec lineages lacked *ompC* mutations but harbored point mutations in the OmpF outer membrane protein (e.g., N80Q, V81I, A83G, and S89N).

**Fig 4:**
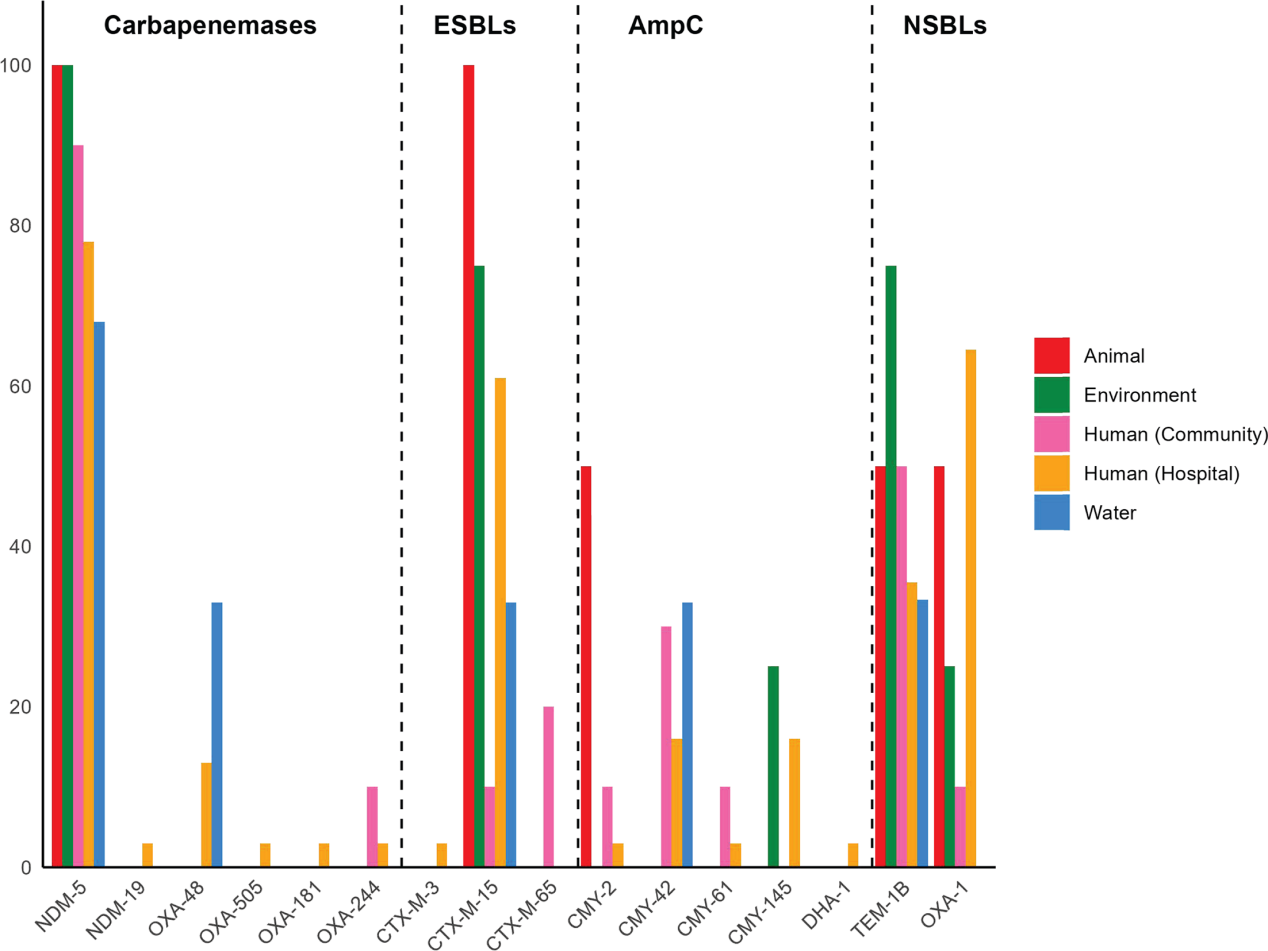
Distribution of β-lactamase genes among carbapenemase-producing *Escherichia coli* (CP-Ec) isolates (*n* = 50) stratified by sampling source. Isolates were obtained from human (community and clinical), animal, water, and environmental sources. Detected β-lactamase genes were categorized into four groups: carbapenemases, extended-spectrum β-lactamases (ESBLs), plasmid-encoded cephalosporinases (AmpC), and narrow-spectrum β-lactamases (NSBLs).

All isolates co-harbored ESBL and/or plasmid-encoded cephalosporinase (AmpC) genes. ESBL genes such as *bla*_CTX-M-15_ (56%, *n*  =  28/50), *bla*_CTX-M-65_ (4%), and *bla*_CTX-M-3_ (2%) were identified. Plasmid-mediated AmpC β-lactamases conferring resistance to extended-spectrum cephalosporins were commonly observed, including *bla*_CMY-2_ (6%), *bla*_CMY-42_ (20%), *bla*_CMY-61_ (2%), *bla*_CMY-145_ (10%), and *bla*_DHA-1_ (8%) (Fig. 4). Moreover, the narrow-spectrum β-lactamase (NSBL) genes *bla*_TEM-1_ and *bla*_OXA-1_ were detected in 38% and 42% of the isolates, respectively.

Beyond β-lactam resistance, CP-Ec isolates frequently carried resistance determinants to aminoglycosides (49/50, 98%), sulfonamides (92%), tetracyclines (76%), and amphenicols (34%), with multiple genes contributing to a multifactorial resistance profile (Table S8). Fluoroquinolone resistance was observed in all isolates. Plasmid-mediated resistance genes *qnrB* (*n* = 4) and *qnrS1* (*n* = 5) were identified in nine distinct isolates. Additionally, chromosomal mutations associated with fluoroquinolone resistance—*gyrA* (S83L, D87N) and *parC* (S80I)—were concurrently detected in 47 of the 50 fluoroquinolone-resistant isolates. Mutations of unknown significance were found in the *pmrA* and *pmrB* (colistin resistance), *cyaA* and *glpT* (fosfomycin resistance), *nfsA* (nitrofurantoin resistance), and *cirA* (iron uptake) were detected. No statistically significant differences in the prevalence of resistance genes were observed between community- and hospital-derived isolates (*P* > 0.05), except for *bla*_OXA-1_, which was significantly associated with hospital isolates (OR = 32.7, 95% CI = 4.50–356.2, *P* < 0.0001) (Fig. 3 and 4).

### Population structure of CP-Ec isolates

**Fig 5:**
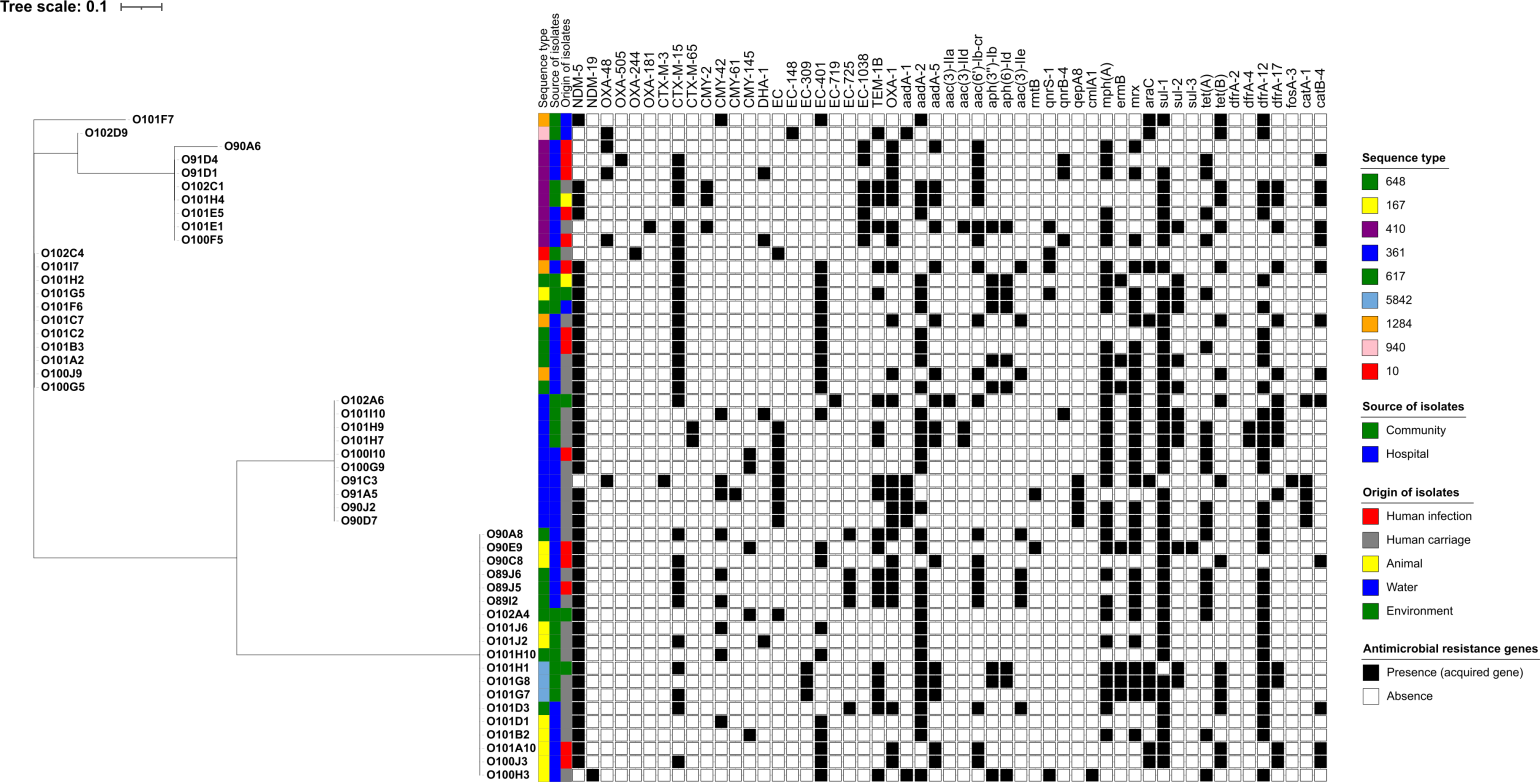
Phylogenetic analysis of carbapenemase-producing *Escherichia coli* (CP-Ec) from community and hospital sources in Lebanon, based on SNP analysis. The legend shows isolation time, sequence types (STs), and antimicrobial resistance genes.

A wide diversity of genetic backgrounds of CP-Ec was observed in the community, with the detection of nine different STs, including ST361 (*n* = 4), ST167 (*n* = 3), ST5842 (*n* = 3), ST410 (*n* = 2), ST617 (*n* = 2), ST648 (*n* = 2), ST10 (*n* = 1), ST940 (*n* = 1), and ST1284 (*n* = 1). Thirty-one additional clinical CP-Ec strains isolated from Lebanese hospitalized patients included ST167 (*n* = 7), ST361 (*n* = 6), ST410 (*n* = 6), ST617 (*n* = 4), ST648 (*n* = 5), and ST1284 (*n* = 3) (Fig. 5).

**Fig 6:**
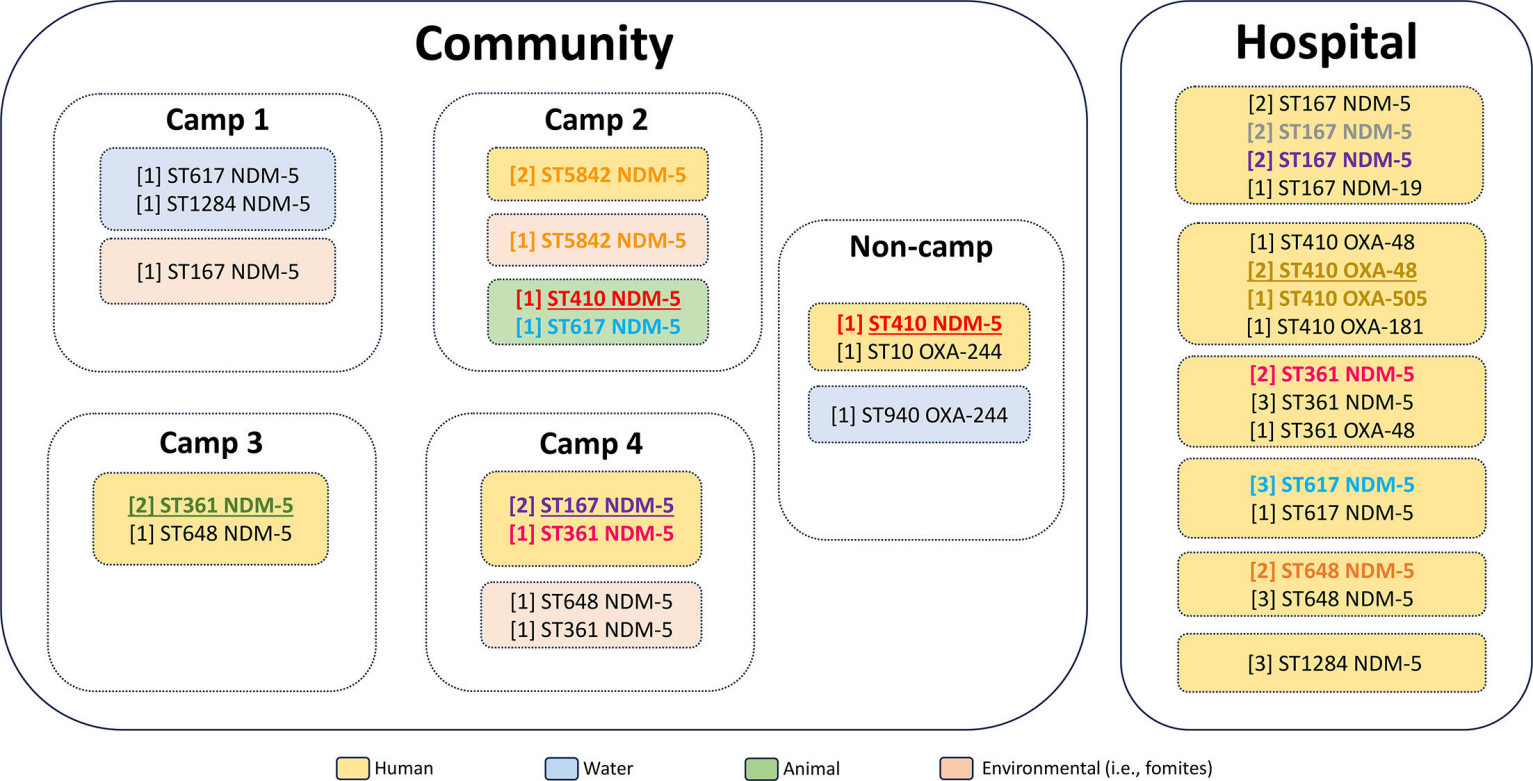
Schematic representation of the distribution of carbapenemase-producing *Escherichia coli* (CP-Ec) across community and hospital settings. Isolates are identified by sample type, sequence type (ST), and carbapenemase gene. Yellow boxes represent CP-Ec-positive individuals, grouped by ST. Blue, green, and pink boxes represent CP-Ec-positive water, animal, and environmental samples, respectively. Genetically related clones are shown in bold and color-coded. Groups of strains with fewer than 50 SNP differences (i.e., genetically identical clones) are highlighted in bold and assigned the same color, with each group using a distinct color. Strains with fewer than 10 SNP differences are additionally underlined.

Pairwise single-nucleotide polymorphism (SNP) distances calculated from core-genome alignments revealed potential dissemination of closely related *bla*_NDM-5_ and *bla*_OXA-48_-carrying strains across diverse hosts and niches within the human-animal-environment continuum (Table S9). Among the nine individuals who tested positive for CP-Ec in the community, several shared the same ST/carbapenemase gene combinations with minimal SNP differences (10–42 SNPs), consistent with recent transmission or a common source. Notably, two Syrian refugees from the same family residing in Camp 3 harbored an ST361/NDM-5 clone differing by only 10 SNPs. Closely related but distinct ST361/NDM-5 clones were also identified in a Syrian refugee in Camp 4 and two unrelated Lebanese hospitalized patients, differing by 30–42 SNPs. In Camp 2, two individuals from the same family carried a genetically similar ST5842/NDM-5 clone, also identified in their surrounding fomites, differing by 26–32 SNPs. Further evidence of interspecies dissemination was observed with ST410/NDM-5 isolates from chicken in Camp 2 and a Lebanese individual in non-camp settings, which differed by only 5 SNPs. Similarly, ST617/NDM-5 clones showing close genetic relatedness were identified in a cow from the same camp and in three different Lebanese hospitalized patients, differing by 38–41 SNPs. In Camp 4, two Syrian refugees from different families carried a closely related ST167/NDM-5 clone (6 SNPs difference), with similar strains also detected in two Lebanese patients in hospital settings (11–22 SNPs difference). Additional closely related clones were found among hospitalized Lebanese patients, including ST648/NDM-5 and ST410/OXA-48 (Fig. 6).

**Fig 7:**
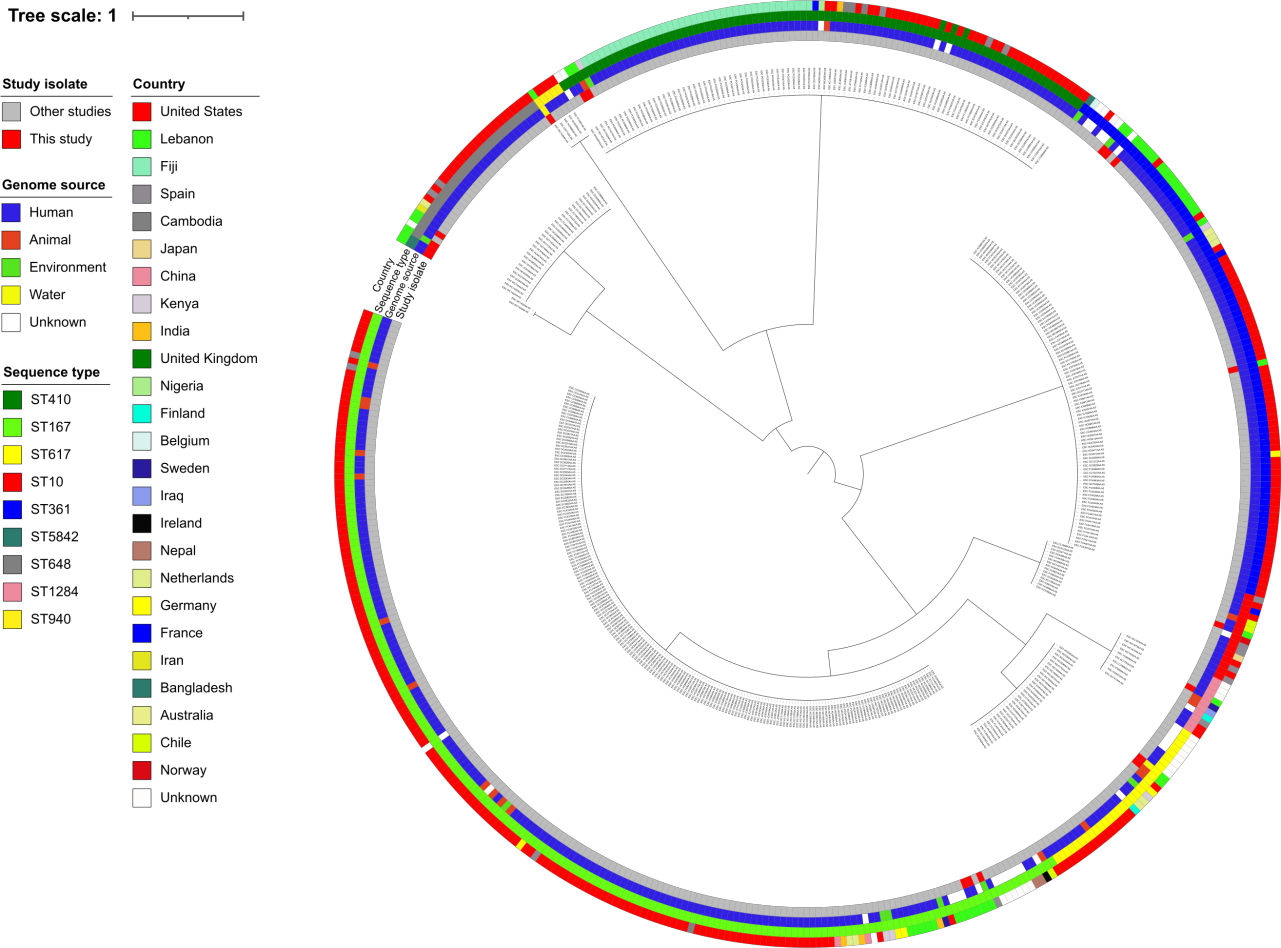
Core genome MLST (cgMLST) phylogenetic tree of carbapenemase-producing *Escherichia coli* (CP-Ec), including 19 community-derived genomes from this study and 447 publicly available genomes (ST10, *n* = 15; ST167, *n* = 189; ST361, *n* = 91; ST410, *n* = 92; ST617, *n* = 31; and ST648, *n* = 30; ST940, *n* = 5; ST1284, *n* = 10; and ST5842, *n* = 3) isolated in 2022. No CP-Ec ST5842 genomes from 2022 were found in EnteroBase. The first ring indicates isolates, the second ring shows genome source (human, animal, environmental, or water), the third ring denotes sequence types (STs), and the fourth ring represents the country of origin.

Pairwise SNP distances derived from cgMLST alignments showed that refugee isolates clustered closely with CP-Ec genomes from multiple countries (Fig. 7). To explore these relationships more deeply within Lebanon (i.e., locally), we focused on the five predominant STs (ST361, ST167, ST617, ST410, and ST648) and generated ST-based maximum likelihood trees incorporating both our isolates and those from previous Lebanese reports. The analysis included 64 additional nonredundant CP-Ec genomes from EnteroBase (Table S4). Notably, ST361 was the most common lineage (*n* = 45), followed by ST167 (*n* = 32) and ST648 (*n* = 17), highlighting their widespread occurrence at the human-animal-environment interface in Lebanon.

**Fig 8:**
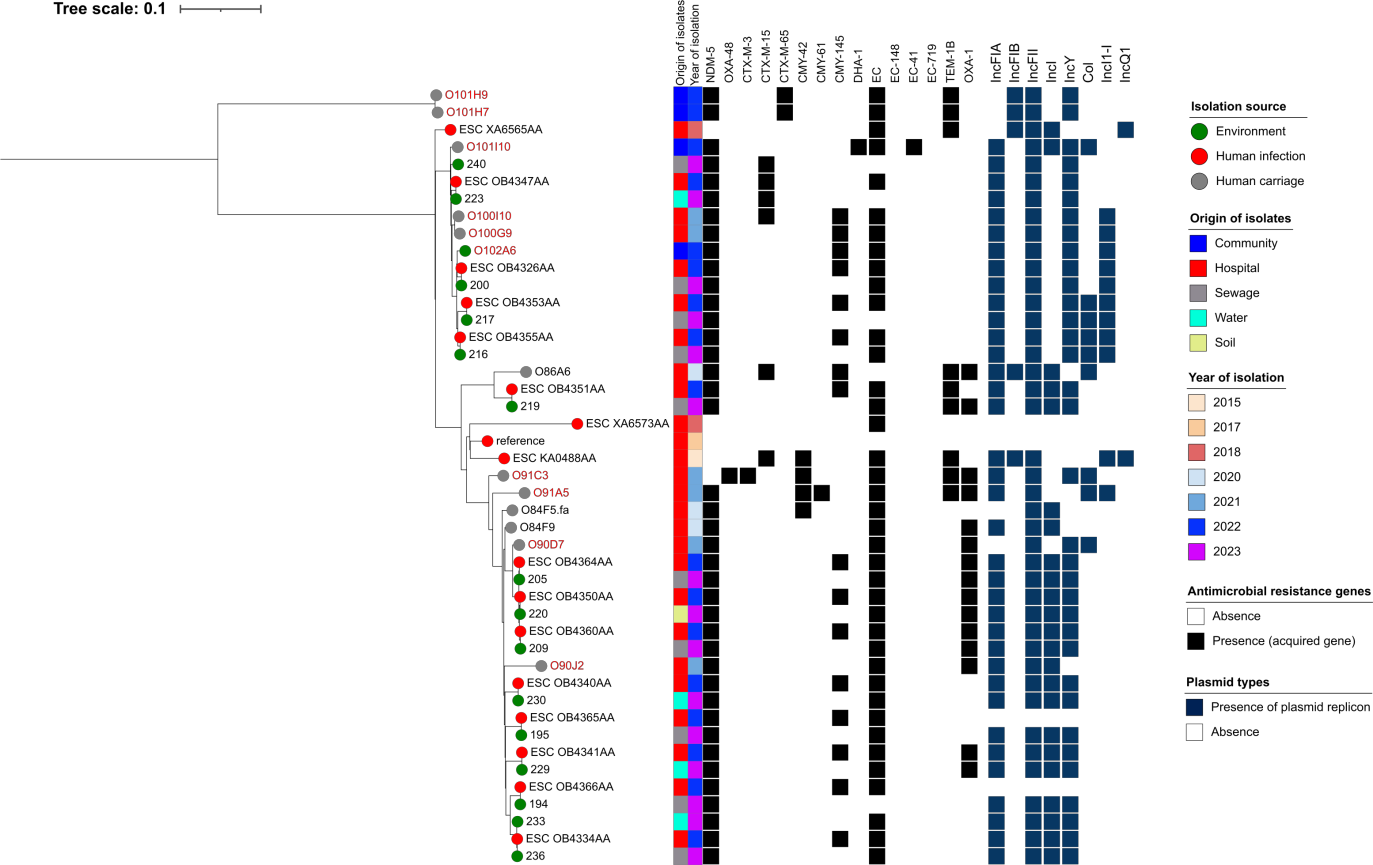
Phylogenetic tree of carbapenemase-producing *Escherichia coli* (CP-Ec) ST361 isolates from Lebanon based on SNP analysis. The legend shows the isolation source (human, animal, water, or environment), time, hospital or community origin, and β-lactam resistance genes; non-β-lactam resistance genes are not shown. Strains labeled in red were collected in this study, whereas those in black were previously isolated in Lebanon and are accessible in public genomic repositories.

Overall, ST361 human and environmental CP-Ec isolates clustered with both clinical and community strains previously reported in Lebanon, all sharing key β-lactam resistance genes. Notably, two isolates from this study—O101I10 (carried by a Syrian refugee) and O102A6 (recovered from fomites), both isolated in Camp 4—were closely related to four clinical strains (ESC OB4326AA, ESC OB4355AA, ESC OB4347AA, and ESC OB4353AA), four environmental strains isolated from sewer water (200, 216, 217, and 240), and one waterborne strain (223) previously reported in Lebanon and retrieved from EnteroBase. Despite differences in source, geographic location, and collection time points (2020–2023), these isolates differed by only 31–56 SNPs. Another cluster of closely related ST361 isolates included one clinical isolate from this study (O90D7), three environmental isolates from sewer water (205 and 209) and soil (220), and three clinical strains retrieved from EnteroBase (ESC_OB4350AA, ESC_OB4360AA, and ESC_OB4364AA), with SNP differences ranging from 33 to 36 (Fig. 8).

**Fig 9:**
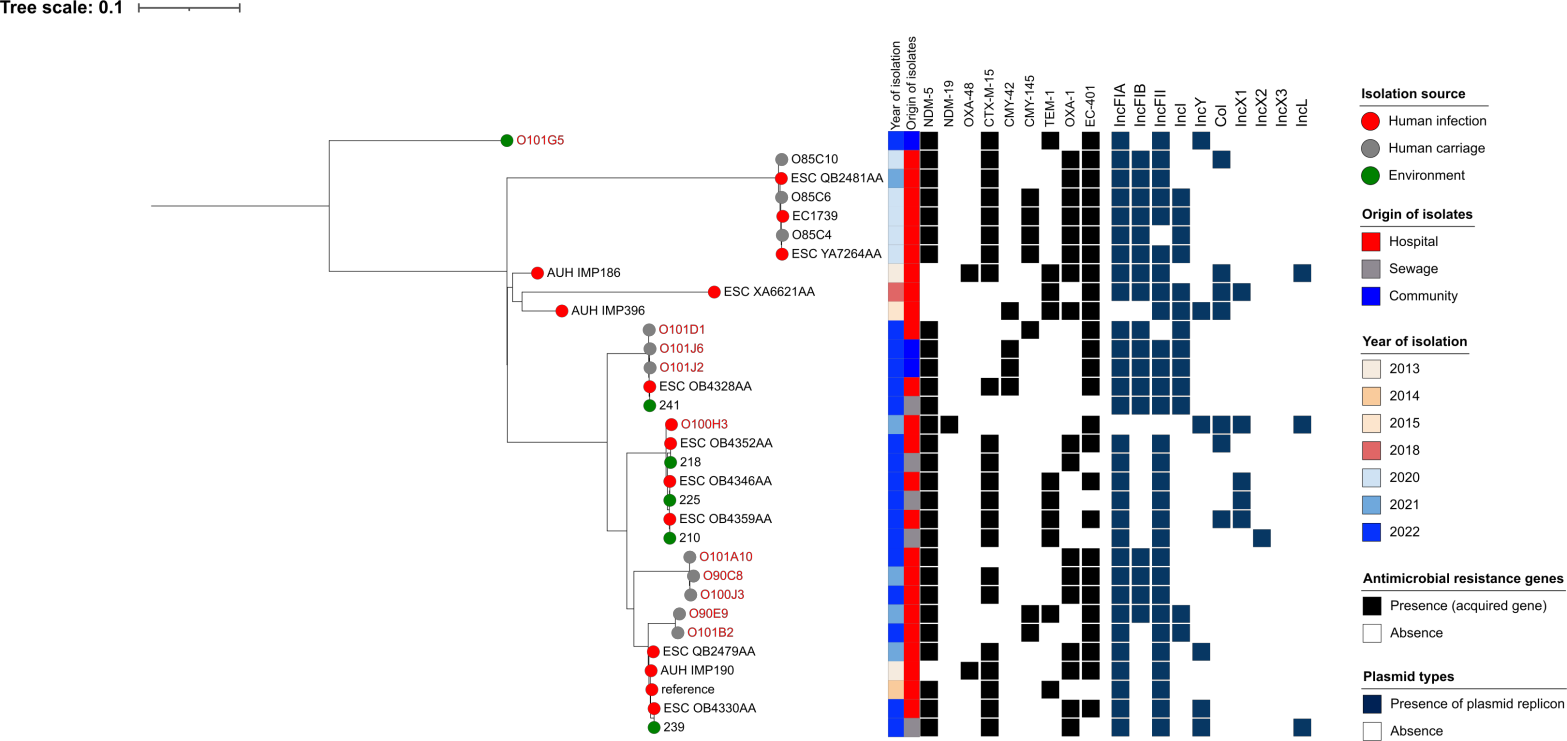
Phylogenetic tree of carbapenemase-producing *Escherichia coli* (CP-Ec) ST167 isolates from Lebanon based on SNP analysis. The legend shows the isolation source (human, animal, water, or environment), time, hospital or community origin, and β-lactam resistance genes; non-β-lactam resistance genes are not shown. Strains labeled in red were collected in this study, whereas those in black were previously isolated in Lebanon and are accessible in public genomic repositories.

Similar clustering patterns were observed for other STs. For example, three ST167 isolates from this study—O101D1 (clinical), O101J6 (community), and O101J2 (community)—clustered with a clinical strain reported in 2022 (ESC OB4328AA) and an environmental strain from sewage collected in 2023 (241), showing 0–21 SNP differences. All five isolates carried both *bla*_NDM-5_ and *bla*_EC-402_. (Fig. 9).

**Fig 10:**
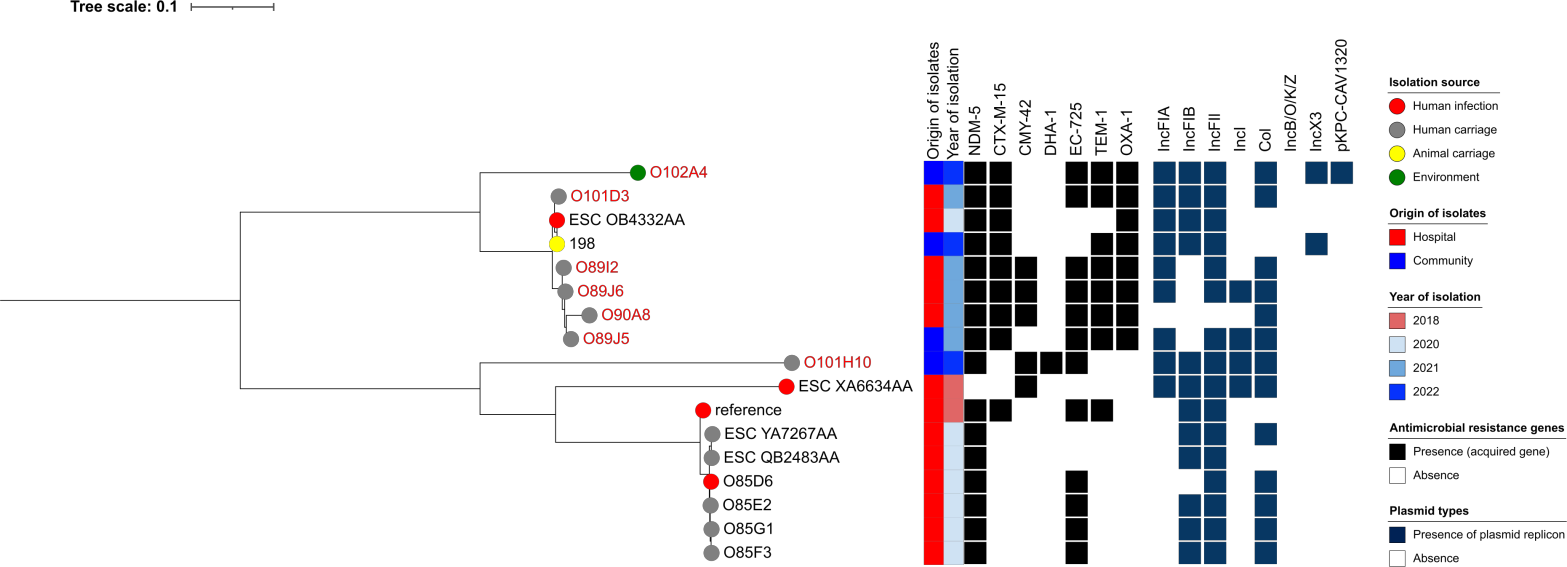
Phylogenetic tree of carbapenemase-producing *Escherichia coli* (CP-Ec) ST648 isolates from Lebanon based on SNP analysis. The legend shows the isolation source (human or environment), time, hospital or community origin, and β-lactam resistance genes; non-β-lactam resistance genes are not shown. Strains labeled in red were collected in this study, whereas those in black were previously isolated in Lebanon and are accessible in public genomic repositories.

**Fig 11:**
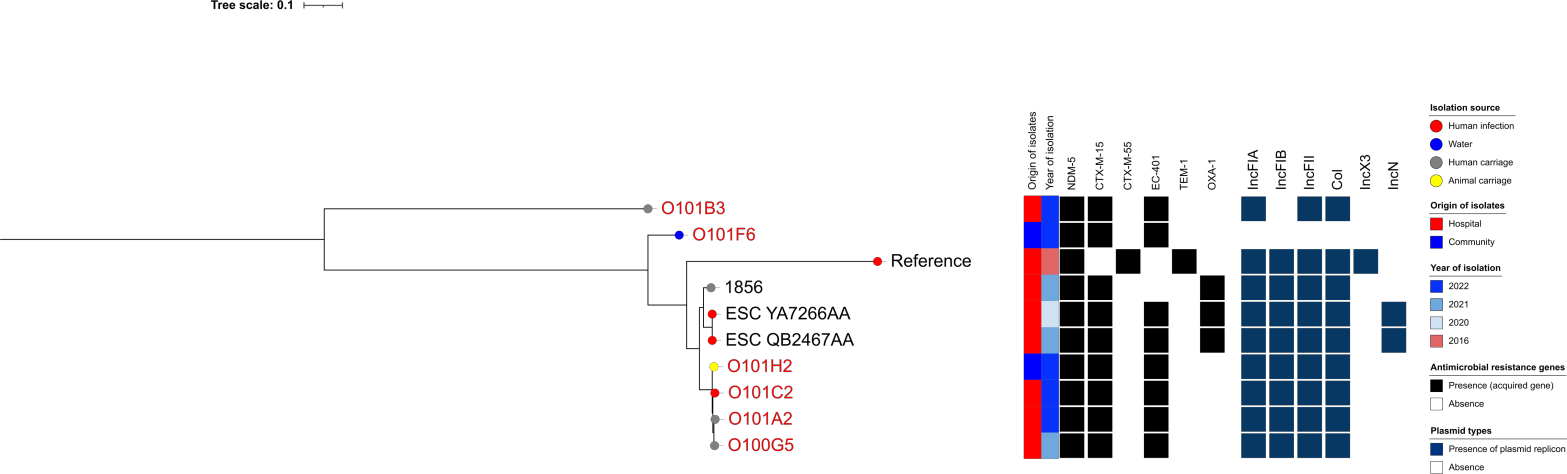
Phylogenetic tree of carbapenemase-producing *Escherichia coli* (CP-Ec) ST617 isolates from Lebanon based on SNP analysis. The legend shows the isolation source (human, animal, water, or environment), time, hospital or community origin, and β-lactam resistance genes; non-β-lactam resistance genes are not shown. Strains labeled in red were collected in this study, whereas those in black were previously isolated in Lebanon and are accessible in public genomic repositories.

**Fig 12:**
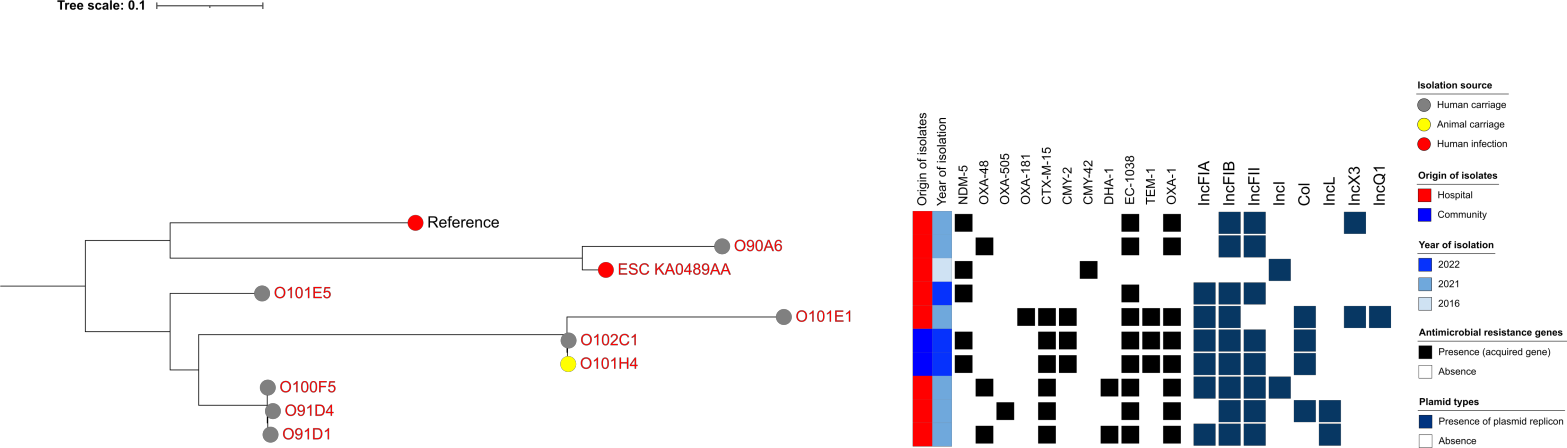
Phylogenetic tree of carbapenemase-producing *Escherichia coli* (CP-Ec) ST410 isolates from Lebanon based on SNP analysis. The legend shows the isolation source (human, animal, water, or environment), time, hospital or community origin, and β-lactam resistance genes; non-β-lactam resistance genes are not shown. Strains labeled in red were collected in this study, whereas those in black were previously isolated in Lebanon and are accessible in public genomic repositories.

Among ST648 isolates, O101D3—a clinical isolate from a hospitalized Lebanese patient in this study—clustered with a clinical strain (ESC_OB4332AA) with 0 SNP difference and with an isolate recovered from an animal source (198) previously reported in Lebanon from EnteroBase, which differed by 56 SNPs (Fig. 10). With respect to ST617—a lineage closely related to ST167—strain O101H2, recovered from cattle in Camp 2, was closely related to three clinical strains isolated from hospitalized patients (O101C2, O101A2, and O100G5), with SNP differences ranging from 33 to 41. These four strains were also related to two clinical strains previously reported in Lebanon (ESC_YA7266AA and ESC_QB2467AA), differing by 243 SNPs. All six strains carried *bla*_NDM-5_*, bla*_CTX-M-15_, and *bla*_EC-401_ (Fig. 11). In contrast, all CP-Ec ST410 isolates recovered from community settings were genetically distinct from those found in clinical settings, both in this study and in previously reported Lebanese data (Fig. 12).

### Plasmid content in CP-Ec isolates

A total of 13 different plasmid replicons were identified in the sequenced isolates. Fifty-six percent of the isolates (28 out of 50) contained at least one plasmid replicon, with a median of two plasmid replicons per isolate. The plasmid replicons IncFII and Col (7/19 each, 37%) were predominant in community sample sources, whereas IncFIA (25/31, 81%) was dominant in clinical samples and had a lower prevalence in the community (6%). No IncFIB, IncQ1, IncN, or ColKP3 plasmids were detected among community isolates. Likewise, no IncB plasmids were detected among clinical isolates.

## DISCUSSION

AMR is a quintessential global health challenge that transcends borders, disproportionately burdening the most vulnerable. Although high-income countries often dominate AMR discourse and surveillance networks, disenfranchised communities in LMICs, particularly those affected by conflict, displacement, and poverty, are increasingly recognized as unmonitored hotspots where AMR can silently proliferate (3). Lebanon exemplifies a plethora of vulnerabilities, including prolonged economic instability, political unrest, debilitated infrastructure, pollution, and a high refugee-to-host population ratio, which have severely strained its healthcare capacity and public health response (4, 5).

To our knowledge, this is the most comprehensive genomic-based study to date investigating carbapenem resistance in a conflict-affected region, and the first to explore its spread through the human-animal-environment interface in both refugee and host communities in resource-limited settings. By integrating CP-Ec isolates from hospitalized patients, underprivileged communities, animals, and environmental sources, we uncovered a wide distribution of globally emerging high-risk clones, predominated by *bla*_NDM-5_-harboring strains with extensive resistance determinants and genetic features of clinical concern. Indeed, our data indicate a notably high carriage prevalence of CP-Ec (10.5%) among Syrian refugees, exceeding previously reported rates in the Lebanese host community within the same geographic region (9). This elevated colonization rate is particularly alarming, given that none of the recruited participants reported antimicrobial use in the 6 months preceding sampling. Importantly, participants with recent infections or antimicrobial exposure were excluded from this study because our primary focus was on community carriage rather than exposure to recent antimicrobial selection pressure clinically, which can favor the emergence of carbapenemase-producing strains in treated individuals (14). These excluded participants might be more likely to harbor carbapenemase genes and exhibit higher rates of AMR, suggesting that the true burden of CP-Ec in these communities could be even higher than our observations. These findings suggest that the dissemination of CP-Ec in these communities is driven not primarily by recent antimicrobial exposure but likely by other factors such as environmental contamination, close interpersonal contact, and inadequate infection prevention and sanitation measures (15). This hypothesis is further supported by the widespread detection of *bla*_NDM-5_-harboring *E. coli* strains in diverse environmental sources (16–19).

Further compounding this concern, all CP-Ec isolates from both community and hospital settings carried ESBL or AmpC genes alongside chromosomal mutations, including alterations in *ftsI*, that confer reduced susceptibility to last-resort and newly developed β-lactam agents, including ceftazidime-avibactam and cefiderocol, despite none of these antimicrobials being in clinical use in Lebanon (16). The isolates remained fully susceptible to only three antimicrobials: neomycin (MIC  ≤  4  µg/mL), apramycin (MIC  ≤  16  µg/mL), and colistin (MIC  ≤  0.5  µg/mL). Of these, neomycin and apramycin are restricted to veterinary use, whereas colistin is increasingly used in clinical settings in low-resource countries, including Lebanon, particularly in response to the growing prevalence of MDR infections (20). Although resistance to colistin was not identified in this study, our previous investigations have documented the spread of plasmid-mediated colistin resistance (*mcr* genes) across Lebanon’s human, animal, and environmental sectors in both refugee and non-refugee settings (9, 21–25). These findings emphasize the precariousness of relying on colistin as a last-resort therapeutic option, especially in contexts where MDR organisms are prevalent, treatment alternatives are limited, and appropriate routine colistin susceptibility testing is not implemented in Lebanese clinical laboratories (23, 26). Among the remaining antimicrobials, only aztreonam-avibactam, amikacin, tigecycline, eravacycline, nitrofurantoin, and fosfomycin demonstrated susceptibility rates above 90%. However, most of these agents are primarily indicated for urinary tract infections and/or may have limited efficacy for treating invasive infections. This highlights the critical need to preserve their efficacy and mitigate resistance development through robust antimicrobial stewardship and continued surveillance efforts, and other interventions that target the socioeconomic status of the population.

Adding to the urgency of our findings, CP-Ec was detected not only in Syrian refugees but also in animals, fomites, and water sources directly linked to refugee environments. This wide distribution underscores the risk of a sustained transmission within camps and potential spillovers into surrounding communities. Interestingly, the refugee isolates clustered with global CP-Ec genomes from multiple countries, demonstrating their circulation in geographically diverse sources rather than representing strictly localized sources. Additionally, they also carried the same resistance genes and exhibited resistance patterns indistinguishable from those found in Lebanese individuals in non-camp settings and hospitalized patients, indicating a shared reservoir of CP-Ec across community and clinical settings. These results support our previous pilot study (18), which documented the emergence of *bla*_NDM-5_-harboring *E. coli* ST617 among otherwise healthy Syrian refugees, their livestock, and the surrounding environment. Beyond ST617, our study identified several CP-Ec lineages, specifically ST167, ST361, ST648, and ST410, from human, animal, and environmental samples in both community and healthcare settings. To further support the evidence of cross-sectoral transmission, we analyzed CP-Ec genomes from EnteroBase and recent studies in Lebanon (7, 17, 27). All public genomes belonged to the same STs as those in our study. Comparative genomic analyses revealed very low pairwise SNP distances and highly similar resistomes, especially among ST167 and ST361. These findings strongly indicate recent transmission events or a shared source, confirming their role in cross-sectoral AMR dissemination (28–32). Globally, these two lineages have emerged as high-risk clones and are now increasing in prevalence across nearly all continents. Both ST167 and ST361 have been reported at the human-animal-environment interface and are now frequently associated with carbapenemase genes, particularly *bla*_NDM-5_ (33–36). Collectively, these findings underscore the broader public health implications of our study. The rapid global expansion of ST167 is particularly concerning. Initially detected in Nepal and France, ST167 clones have since disseminated across Southeast Asia and Europe and have been identified in the United States, frequently exhibiting high genomic similarity to European isolates (37). Between 2017 and 2019, the spread of ST167 CP-Ec was also documented in Israel, where it replaced ST410 as the dominant lineage, a shift believed to have originated from strains introduced from the United States. Reports of ST167 clones have also emerged from Africa and Australia, further illustrating their broad geographic reach and ecological versatility (37, 38). Indeed, a recent multi-country study involving 13 European Union/European Economic Area nations between 2012 and 2022 identified ST167 as the most common CP-Ec lineage (23%), whereas ST361, although less frequent overall (8%), was notably most common in France (19.7%), Switzerland (15.5%), Thailand (11.4%), and India (10.7%) (29).

Both ST167 and ST361 are characterized by substantial genetic plasticity, enabling the acquisition of additional resistance and virulence determinants that enhance their fitness in diverse ecological niches. Their concurrent detection in our study across human, animal, and environmental reservoirs, within refugee settlements and underserved host communities, raises critical concerns. In a region marked by fragile infrastructure, limited AMR surveillance, and high population mobility, these lineages may serve as reservoirs and transmission hubs with potential for wider regional and global spread. Importantly, all isolates in this study were predicted to be potential human pathogens with high probability (>95%), carrying multiple virulence-associated factors (Table S10), further elevating concerns about their capacity to cause severe infections. Notably, although ST167 and ST361 strains lacked the *fyuA* gene in the EU/EEA multi-country investigation (37), our bioinformatic analysis revealed the presence of this key iron-acquisition virulence factor in these and other STs (Table S11), highlighting enhanced pathogenic potential.

This study reveals a real public health threat affecting both refugee populations and marginalized host communities, which together form niches conducive to the emergence, amplification, and transmission of MDR organisms. Furthermore, the presence of CP-Ec strains in animals, water, and environmental surfaces suggests that community-acquired infections may originate from sources traditionally considered low-risk, extending the threat beyond hospital walls. The convergence of resistance and virulence, coupled with the presence of nearly identical high-risk clones across community and hospital interfaces, illustrates both environmental persistence and sustained local transmission of MDR *E. coli*. Notably, underprivileged communities, often excluded from global genomic data sets, are tightly interconnected with international mobility, trade, and healthcare systems, rendering local resistance threats a potential catalyst for global crises. Overall, we predict that transmission happens between refugee and hosting populations, likely in both directions. We also predict that deteriorating conditions have rendered these populations susceptible to environmental contamination, which has likely resulted in a cycle of evolution, emergence, and spread of AMR between refugees, the hosting community, and the environment. Taken together, our observations reinforce the paramount need for a coordinated and comprehensive One Health approach to AMR surveillance and mitigation. Efforts must integrate enhanced genomic surveillance, infection prevention, and antimicrobial stewardship across public health, veterinary, and environmental sectors. Such integrated interventions are critical to halting further dissemination and preserving the effectiveness of the few remaining treatment options, particularly in regions where infectious disease burdens and AMR risks are high, and healthcare resources remain scarce.

Interestingly, the return of refugees to Syria following the collapse of the Assad regime in December 2024 presents a significant public health concern, with the potential to accelerate the regional and global spread of AMR. Through our studies (8, 18), we confirmed that many returnees are likely to carry MDR pathogens, further complicating containment efforts. Syria’s healthcare system remains severely debilitated by years of conflict, with minimal diagnostic capacity, limited access to effective antimicrobials, and no functional national AMR surveillance program (39). These challenges are compounded by widespread destruction of sanitation infrastructure and wastewater treatment facilities, increasing the risk of environmental dissemination of MDR organisms through contaminated water sources (40). Ongoing internal displacement and sustained contact with diaspora communities in the Gulf region, Europe, and North America further facilitate the silent spread of resistance determinants across borders. Without urgent investment in healthcare reconstruction, environmental protection, and robust AMR monitoring, the reintroduction and undetected circulation of MDR *E. coli*, including carbapenemase-producing strains, in Syria could pose an escalating and under-recognized threat to regional and global health security.

This study has several limitations. Although our cross-sectional sampling strategy was designed to capture a wide range of human, animal, and environmental sources, it did not allow for longitudinal follow-up or repeated sampling from the same individuals or sites over time. Additionally, the overall number of samples and confirmed CP-Ec isolates was relatively limited. The study was conducted over a relatively short period of time (July–August 2022), which might have limited our ability to capture seasonal and temporal variations in AMR patterns. As a result, the data may not fully represent Lebanon’s genomic diversity and AMR burden. Our study focused only on CP-Ec and did not assess other carbapenem-resistant Enterobacterales. The use of culture-based screening methods may have limited sensitivity. This limitation may underestimate the true prevalence of carbapenemase-producing *E. coli*, especially *in vitro* ertapenem-susceptible strains, such as those producing *bla*_OXA-244_. We also did not include long-read sequencing, plasmid conjugation assays, or detailed temporal data. This restricted our ability to resolve plasmid structures, assess mobility, and infer transmission direction. These limitations are largely associated with the availability of funding for these studies and the ability to access refugees and collect samples in turbulent and war-stricken areas. Despite these limitations, the study’s strength lies in its integrative One Health approach. We combined isolates from multiple reservoirs and previous studies and applied whole-genome sequencing to analyze resistance patterns in one of the world’s most underserved and crisis-affected regions. Our findings highlight an urgent need for improved AMR surveillance, stronger laboratory capacity, and targeted public health interventions in marginalized communities. These settings are where resistance can silently evolve and spread between humans, animals, and the environment, with global implications.

## CONCLUSION

This study provides critical genomic insights into the silent dissemination of CP-Ec in a conflict-affected, severely under-resourced setting. As previously hypothesized (18, 24), the sources of MDR bacteria in Lebanon have become increasingly diffuse. Our findings confirm the active acquisition and dissemination of globally emerging high-risk *E. coli* lineages, primarily *bla*_NDM-5_-harboring ST167 and ST361, across the human-animal-environment interface. The detection of closely related strains belonging to globally prevalent STs in diverse ecological niches highlights the risk of local amplification and cross-border dissemination. Alarmingly, we identified resistance to last-resort antimicrobials not yet introduced in Lebanon, reflecting growing concerns about the clinical relevance of these drugs and the risk of global transmission of these MDR *E. coli* clones. These results reinforce the urgent need to expand integrated One Health genomic surveillance, particularly in disenfranchised communities. They also draw attention to the overlooked contribution of conflict, displacement, and structural health disparities in accelerating AMR. Without timely, coordinated interventions, these resistance threats will continue to escalate, undermining both regional and global containment efforts.

## Data Availability

The generated genomes have been deposited at DDBJ/ENA/GenBank under the accession number PRJNA1238219.

## SUPPLEMENTAL MATERIAL

Supplemental tables aem.01625-25-s0001.docx Tables S1 to S5, S7, S10, and S11.

Table S6 aem.01625-25-s0002.xlsx Antimicrobial resistance patterns of carbapenemase-producing *Escherichia coli* isolates recovered in this study.

Table S8 aem.01625-25-s0003.xlsx Genomic characterization of carbapenemase-producing *Escherichia coli* isolates collected in this study.

Table S9 aem.01625-25-s0004.xlsx Maximum-likelihood phylogenetic tree and pairwise SNV distance matrix of *Escherichia coli* isolates from this study and all publicly available Lebanese *E. coli* positive genomes.

ASM does not own the copyrights to Supplemental Material that may be linked to, or accessed through, an article. The authors have granted ASM a non-exclusive, world-wide license to publish the Supplemental Material files. Please contact the corresponding author directly for reuse.
