# Supplementary material for: Evidence of transmission and dissemination of diverse blaNDM-5-producing Escherichia coli clones between refugee and host communities and their environment: a multicenter cross-sectional study
Source: Appl Environ Microbiol. 2025 Nov 4;91(12):e01625-25. doi: 10.1128/aem.01625-25 (PMC12724354; doi:10.1128/aem.01625-25)
Supplement: Supplemental tables — Tables S1 to S5, S7, S10, and S11. [file aem.01625-25-s0001.docx]

**S1 Table.** Carbapenemase-Producing *Escherichia coli* in Human and Animal Stool Samples and Environmental Sources: Demographic and Sample Characteristics.

| Sample ID | Family  (#n) | Settings (camp or community) | Sample type | Nationality | Sex | Age (years) | Carbapenemase- harboring  *E. coli* and ST |
| --- | --- | --- | --- | --- | --- | --- | --- |
|  |  |  |  |  |  |  |  |
| A006(S) | 1 | Camp 1 | Human | Syrian | F | 30 | - |
| A007(S) | 1 | Camp 1 | Human | Syrian | M | 6 | - |
| A008(S) | 1 | Camp 1 | Human | Syrian | F | 5 | - |
| A009(S) | 1 | Camp 1 | Human | Syrian | F | 2 | - |
| A010(S) | 1 | Camp 1 | Human | Syrian | M | 33 | - |
| B''015(S) | 2 | Camp 1 | Human | Syrian | M | 9 | - |
| B''017(S) | 2 | Camp 1 | Human | Syrian | F | 2 | - |
| C001(S) | 3 | Camp 1 | Human | Syrian | F | 36 | - |
| C002(S) | 3 | Camp 1 | Human | Syrian | M | 3 | - |
| C003(S) | 3 | Camp 1 | Human | Syrian | F | 2 | - |
| C004(S) | 3 | Camp 1 | Human | Syrian | F | 1 | - |
| C005(S) | 3 | Camp 1 | Human | Syrian | M | 33 | - |
| D''018(S) | 4 | Camp 1 | Human | Syrian | F | 27 | - |
| D''019(S) | 4 | Camp 1 | Human | Syrian | M | 2 | - |
| D''020(S) | 4 | Camp 1 | Human | Syrian | M | 4 | - |
| D''021(S) | 4 | Camp 1 | Human | Syrian | F | 7 | - |
| D''022(S) | 4 | Camp 1 | Human | Syrian | M | 37 | - |
| E''025(S) | 5 | Camp 1 | Human | Syrian | M | 43 | - |
| E''026(S) | 5 | Camp 1 | Human | Syrian | F | 36 | - |
| E''028(S) | 5 | Camp 1 | Human | Syrian | F | 4 | - |
| E''029(S) | 5 | Camp 1 | Human | Syrian | M | 3 | - |
| F030(S) | 6 | Camp 2 | Human | Syrian | M | 40 | - |
| F031(S) | 6 | Camp 2 | Human | Syrian | F | 16 | - |
| F032(S) | 6 | Camp 2 | Human | Syrian | M | 15 | - |
| F033(S) | 6 | Camp 2 | Human | Syrian | F | 13 | - |
| F034(S) | 6 | Camp 2 | Human | Syrian | F | 5 | *bla*_NDM-5_, ST5842 |
| F035(S) | 6 | Camp 2 | Human | Syrian | F | 3 | *bla*_NDM-5_, ST5842 |
| F036(S) | 6 | Camp 2 | Human | Syrian | F | 35 | - |
| G037(S) | 7 | Camp 2 | Human | Syrian | M | 48 | - |
| G038(S) | 7 | Camp 2 | Human | Syrian | F | 15 | - |
| G039(S) | 7 | Camp 2 | Human | Syrian | M | 22 | - |
| G040(S) | 7 | Camp 2 | Human | Syrian | F | 2 months* | - |
| H041(S) | 8 | Camp 3 | Human | Syrian | F | 38 | - |
| H042(S) | 8 | Camp 3 | Human | Syrian | M | 8 | - |
| I044(S) | 9 | Camp 3 | Human | Syrian | F | 36 | - |
| I045(S) | 9 | Camp 3 | Human | Syrian | F | 9 | - |
| I046(S) | 9 | Camp 3 | Human | Syrian | F | 11 | - |
| I047(S) | 9 | Camp 3 | Human | Syrian | M | 1 | - |
| I048(S) | 9 | Camp 3 | Human | Syrian | F | 17 | - |
| J049(S) | 10 | Camp 3 | Human | Syrian | F | 34 | - |
| J050(S) | 10 | Camp 3 | Human | Syrian | F | 14 | - |
| J051(S) | 10 | Camp 3 | Human | Syrian | M | 12 | - |
| J052(S) | 10 | Camp 3 | Human | Syrian | M | 9 | - |
| K053(S) | 11 | Camp 3 | Human | Syrian | M | 45 | - |
| K054(S) | 11 | Camp 3 | Human | Syrian | F | 45 | *bla*_NDM-5_, ST361 |
| K055(S) | 11 | Camp 3 | Human | Syrian | F | 16 | *bla*_NDM-5_, ST361 |
| K056(S) | 11 | Camp 3 | Human | Syrian | F | 39 | - |
| K057(S) | 11 | Camp 3 | Human | Syrian | F | 13 | - |
| K058(U)1 | 11 | Camp 3 | Human | Syrian | F | 10 | *bla*_NDM-5_, ST648 |
| K059(S) | 11 | Camp 3 | Human | Syrian | F | 5 | - |
| L060(S) | 12 | Camp 3 | Human | Syrian | M | 30 | - |
| L061(S) | 12 | Camp 3 | Stool | Syrian | F | 43 | - |
| M062(S) | 13 | Camp 3 | Human | Syrian | F | 50 | - |
| M063(S) | 13 | Camp 3 | Human | Syrian | F | 8 | - |
| M064(S) | 13 | Camp 3 | Human | Syrian | F | 17 | - |
| N064(S) | 14 | Camp 4 | Human | Syrian | M | 42 | - |
| N065(S) | 14 | Camp 4 | Human | Syrian | F | 33 | - |
| N066(S) | 14 | Camp 4 | Human | Syrian | M | 7 | - |
| N067(S) | 14 | Camp 4 | Human | Syrian | F | 4 | - |
| N068(S) | 14 | Camp 4 | Human | Syrian | F | 3 | *bla*_NDM-5_, ST361 |
| O069(S) | 15 | Camp 4 | Human | Syrian | F | 27 | - |
| O070(S) | 15 | Camp 4 | Human | Syrian | M | 39 | - |
| O071(S) | 15 | Camp 4 | Human | Syrian | M | 3 | *bla*_NDM-5_, ST167 |
| O072(S) | 15 | Camp 4 | Human | Syrian | F | 7 | - |
| O073(S) | 15 | Camp 4 | Human | Syrian | F | 8 | - |
| O074(S) | 15 | Camp 4 | Human | Syrian | F | 5 | - |
| O075(S) | 15 | Camp 4 | Stool | Syrian | F | 7 | - |
| P075(S) | 16 | Camp 4 | Human | Syrian | F | 56 | - |
| Q076(S) | 17 | Camp 4 | Human | Syrian | F | 50 | *bla*_NDM-5_, ST167 |
| Q077(N) | 17 | Camp 4 | Stool | Syrian | F | 20 | - |
| R079(S) | 18 | Camp 4 | Human | Syrian | M | 22 | - |
| R080(S) | 18 | Camp 4 | Human | Syrian | F | 50 | - |
| R082(S) | 18 | Camp 4 | Human | Syrian | M | 2 | - |
| LA001(S) | 19 | Non-camp | Human | Lebanese | M | 58 | - |
| LA002(S) | 19 | Non-camp | Human | Lebanese | F | 43 | - |
| LA003(S) | 19 | Non-camp | Human | Lebanese | F | 16 | - |
| LA004(S) | 19 | Non-camp | Human | Lebanese | M | 13 | - |
| LA005(S) | 19 | Non-camp | Human | Lebanese | F | 4 | - |
| LA006(S) | 19 | Non-camp | Human | Lebanese | M | 50 | - |
| LB007(S) | 20 | Non-camp | Human | Lebanese | F | 47 | - |
| LB008(S) | 20 | Non-camp | Human | Lebanese | M | 52 | - |
| LB009(S) | 20 | Non-camp | Human | Lebanese | F | 47 | - |
| LC010(S) | 21 | Non-camp | Human | Lebanese | M | 52 | - |
| LC011(S) | 21 | Non-camp | Human | Lebanese | M | 14 | - |
| LD013(S) | 22 | Non-camp | Human | Lebanese | F | 66 | - |
| LD014(S) | 22 | Non-camp | Human | Lebanese | F | 57 | *bla*_NDM-5_, ST410 |
| LD015(S) | 22 | Non-camp | Human | Lebanese | M | 22 | - |
| LE016(S) | 23 | Non-camp | Human | Lebanese | F | 23 | - |
| LE017(S) | 23 | Non-camp | Human | Lebanese | F | 46 | - |
| SA001(S) | 24 | Non-camp | Human | Syrian | F | 12 | - |
| SA002(S) | 24 | Non-camp | Human | Syrian | M | 14 | - |
| SA003(S) | 24 | Non-camp | Human | Syrian | M | 38 | *bla*_OXA-244_, ST10 |
| SA004(S) | 24 | Non-camp | Human | Syrian | M | 10 | - |
| SB006(S) | 24 | Non-camp | Human | Syrian | F | 6 | - |
| SB007(S) | 24 | Non-camp | Human | Syrian | M | 8 | - |
| A001(W) | 1 | Camp 1 | Drinking water |  |  |  | - |
| B"002(W) | 2 | Camp 1 | Drinking water |  |  |  | - |
| C003(W) | 3 | Camp 1 | Drinking water |  |  |  | - |
| D''004(W) | 4 | Camp 1 | Drinking water |  |  |  | - |
| E''005(W) | 5 | Camp 1 | Drinking water |  |  |  | *bla*_NDM-5_, ST617 |
| KL(W)001 | NA** | Camp 1 | River |  |  |  | *bla*_NDM-5_, ST1284 |
| KL(W)003 | NA** | Camp 1 | Drinking water |  |  |  | - |
| KL(W)004 | NA** | Camp 1 | Drinking water |  |  |  | - |
| KL(W)005 | NA** | Camp 1 | Drinking water |  |  |  | - |
| KL(W)008 | NA** | Camp 1 | Drinking water |  |  |  | - |
| KL(W)009 | NA** | Camp 1 | Drinking water |  |  |  | - |
| KL(W)010 | NA** | Camp 1 | Drinking water |  |  |  | - |
| KL(W)011 | NA** | Camp 1 | Drinking water |  |  |  | - |
| KL(W)012 | NA** | Camp 1 | Drinking water |  |  |  | - |
| KL(W)013 | NA** | Camp 1 | Drinking water |  |  |  | - |
| KL(W)014 | NA** | Camp 1 | Drinking water |  |  |  | - |
| KL(W)015 | NA** | Camp 1 | Drinking water |  |  |  | - |
| KL(W)016 | NA** | Camp 1 | Drinking water |  |  |  | - |
| KL(W)017 | NA** | Camp 1 | Drinking water |  |  |  | - |
| KL(W)018 | NA** | Camp 1 | Drinking water |  |  |  | - |
| KL(W)019 | NA** | Camp 1 | Drinking water |  |  |  | - |
| KL(W)020 | NA** | Camp 1 | Drinking water |  |  |  | - |
| KL(W)021 | NA** | Camp 1 | Drinking water |  |  |  | - |
| KL(W)022 | NA** | Camp 1 | Drinking water |  |  |  | - |
| MI023(W) | NA** | Camp 2 | River |  |  |  | - |
| F031(W) | 6 | Camp 2 | Drinking water |  |  |  | - |
| F032(W)k | 6 | Camp 2 | Drinking water |  |  |  | - |
| F033(W) | 6 | Camp 2 | Drinking water |  |  |  | - |
| G037(W) | 7 | Camp 2 | Drinking water |  |  |  | - |
| G038(W) | 7 | Camp 2 | Drinking water |  |  |  | - |
| H041(W)1 | 8 | Camp 3 | Drinking water |  |  |  | - |
| H041(W)2 | 8 | Camp 3 | Drinking water |  |  |  | - |
| I044(W)1 | 9 | Camp 3 | Drinking water |  |  |  | - |
| I044(W)2 | 9 | Camp 3 | Drinking water |  |  |  | - |
| J049(W)1 | 10 | Camp 3 | Drinking water |  |  |  | - |
| J049(W)2 | 10 | Camp 3 | Drinking water |  |  |  | - |
| K053(W)2 | 11 | Camp 3 | Drinking water |  |  |  | - |
| M062(W)1 | 13 | Camp 3 | Drinking water |  |  |  | - |
| M062(W)2 | 13 | Camp 3 | Drinking water |  |  |  | - |
| N064(W) | 14 | Camp 3 | Drinking water |  |  |  | - |
| N065(W) | 14 | Camp 3 | Drinking water |  |  |  | - |
| O069(W) | 15 | Camp 3 | Drinking water |  |  |  | - |
| O070(W) | 15 | Camp 3 | Drinking water |  |  |  | - |
| P075(W) | 16 | Camp 3 | Drinking water |  |  |  | - |
| Q076(W) | 17 | Camp 3 | Drinking water |  |  |  | - |
| R081(W) | 18 | Camp 3 | Drinking water |  |  |  | - |
| MH024(W) | NA** | Camp 4 | Drinking water |  |  |  | - |
| MH025(W)k | NA** | Camp 4 | Drinking water |  |  |  | - |
| MH025(W) | NA** | Camp 4 | Drinking water |  |  |  | - |
| MH026(W) | NA** | Camp 4 | Drinking water |  |  |  | - |
| LA003(W)d | 19 | Non-camp | Drinking water |  |  |  | *bla*_OXA-48_, ST940 |
| LB007(W)k | 20 | Non-camp | Drinking water |  |  |  | - |
| LC010(W)k | 21 | Non-camp | Drinking water |  |  |  | - |
| LD014(W)d | 22 | Non-camp | Drinking water |  |  |  | - |
| SA001(W)d | 24 | Non-camp | Drinking water |  |  |  |  |
| SB005(W)d | 24 | Non-camp | Drinking water |  |  |  |  |
| SB009(W)d | 24 | Non-camp | Drinking water |  |  |  | - |
| MI001(A-C) | NA** | Camp 2 | Cow |  |  |  | *bla*_NDM-5_, ST617 |
| MI002(A-G) | NA** | Camp 2 | Goat |  |  |  | - |
| MI003(A-Sh1) | NA** | Camp 2 | Sheep |  |  |  | - |
| MI004(A-Sh2) | NA** | Camp 2 | Sheep |  |  |  | - |
| F003(A-Ch) | 6 | Camp 2 | Chicken |  |  |  | - |
| F007(A-Ch) | 6 | Camp 2 | Chicken |  |  |  | - |
| G001(A-Ch) | 7 | Camp 2 | Chicken |  |  |  | - |
| G002(A-Ch) | 7 | Camp 2 | Chicken |  |  |  | *bla*_NDM-5_, ST410 |
| KL001 | NA** | Camp 1 | Fomite |  |  |  | - |
| KL002 | NA** | Camp 1 | Fomite |  |  |  | - |
| KL003 | NA** | Camp 1 | Fomite |  |  |  | - |
| KL008 | NA** | Camp 1 | Fomite |  |  |  | - |
| KL013 | NA** | Camp 1 | Fomite |  |  |  | - |
| KL014 | NA** | Camp 1 | Fomite |  |  |  | - |
| KL015(E)1 | NA** | Camp 1 | Fomite |  |  |  | - |
| KL016 | NA** | Camp 1 | Fomite |  |  |  | - |
| KL017 | NA** | Camp 1 | Fomite |  |  |  | - |
| KL018(E)2 | NA** | Camp 1 | Fomite |  |  |  | - |
| KL019( E ) | NA** | Camp 1 | Fomite |  |  |  | - |
| KL022( E ) | NA** | Camp 1 | Fomite |  |  |  | - |
| E''025(E)3 | 5 | Camp 1 | Fomite |  |  |  | - |
| E''025( E ) | 5 | Camp 1 | Fomite |  |  |  | - |
| KL001(T) | NA** | Camp 1 | Fomite |  |  |  | - |
| KL002(T)2 | NA** | Camp 1 | Fomite |  |  |  | - |
| KL003(T) | NA** | Camp 1 | Fomite |  |  |  | - |
| KL004 (T) | NA** | Camp 1 | Fomite |  |  |  | - |
| KL005(T) | NA** | Camp 1 | Fomite |  |  |  | - |
| KL007(T) | NA** | Camp 1 | Fomite |  |  |  | - |
| KL008(T) | NA** | Camp 1 | Fomite |  |  |  | - |
| KL009(T) | NA** | Camp 1 | Fomite |  |  |  | *bla*_NDM-5_, ST167 |
| KL011(T) | NA** | Camp 1 | Fomite |  |  |  | - |
| KL013(T) | NA** | Camp 1 | Fomite |  |  |  | - |
| KL015(T) | NA** | Camp 1 | Fomite |  |  |  | - |
| KL019(T) | NA** | Camp 1 | Fomite |  |  |  | - |
| KL020(T) | NA** | Camp 1 | Fomite |  |  |  | - |
| E''022(T) | 5 | Camp 1 | Fomite |  |  |  | - |
| F031(E) | 6 | Camp 2 | Fomite |  |  |  | *bla*_NDM-5_, ST5842 |
| G037(T) | 7 | Camp 2 | Fomite |  |  |  | - |
| K053 | 11 | Camp 3 | Fomite |  |  |  | - |
| L060 | 12 | Camp 3 | Fomite |  |  |  | - |
| H041(T) | 8 | Camp 3 | Fomite |  |  |  | - |
| J049(T) | 10 | Camp 3 | Fomite |  |  |  | - |
| L060(T) | 12 | Camp 3 | Fomite |  |  |  | - |
| M062(T) | 13 | Camp 3 | Fomite |  |  |  | - |
| N064 | 14 | Camp 4 | Fomite |  |  |  | - |
| N065 | 14 | Camp 4 | Fomite |  |  |  | - |
| O069 | 15 | Camp 4 | Fomite |  |  |  | - |
| N065(T) | 14 | Camp 4 | Fomite |  |  |  | - |
| O069(T) | 15 | Camp 4 | Fomite |  |  |  | - |
| O070(T) | 15 | Camp 4 | Fomite |  |  |  | *bla*_NDM-5_, ST648 |
| P075(T) | 16 | Camp 4 | Fomite |  |  |  | - |
| Q076(T) | 17 | Camp 4 | Fomite |  |  |  |  |
| R079(T) | 18 | Camp 4 | Fomite |  |  |  | *bla*_NDM-5_, ST361 |
| LA001 | 19 | Non-camp | Fomite |  |  |  | - |
| LA002(T) | 19 | Non-camp | Fomite |  |  |  | - |
| LB007 | 20 | Non-camp | Fomite |  |  |  | - |
| SA001 | 24 | Non-camp | Fomite |  |  |  | - |
| SA001(T) | 24 | Non-camp | Fomite |  |  |  | - |
| SB005 | 24 | Non-camp | Fomite |  |  |  | - |
| O03E2 | 15 | Camp 1 | Fomite |  |  |  | - |
| O08E | 15 | Camp 1 | Fomite |  |  |  | - |

* Ages under 1 year are shown in months.

**The sample was obtained from a communal area or water source used by the refugee population in the makeshift camp.

**S2 Table.** Occurrence of carbapenemase-producing *Escherichia coli* (CP-Ec) in human, animal, and environmental samples from Syrian refugees and surrounding host communities across camp and non-camp settings.

| Settings | Source | Population (refugees or host) | #n | Presence of carbapenemase-producing *Escherichia coli* (n) |
| --- | --- | --- | --- | --- |
| Camp 1 | Human | Refugees | 21 | 0 |
|  | Drinking water | Refugees | 23 | 1 |
|  | River | Refugees | 1 | 1 |
|  | Fomites | Refugees | 30 | 1 |
| Camp 2 | Human | Refugees | 11 | 2 |
|  | Animal | Refugees | 8 | 2 |
|  | Drinking water | Refugees | 5 | 0 |
|  | River | Refugees | 1 | 0 |
|  | Fomites | Refugees | 2 | 1 |
| Camp 3 | Human | Refugees | 23 | 3 |
|  | Drinking water | Refugees | 16 | 0 |
|  | Fomites | Refugees | 6 | 0 |
| Camp 4 | Human | Refugees | 18 | 3 |
|  | Drinking water | Refugees | 4 | 0 |
|  | Fomites | Refugees | 9 | 2 |
| Non-camp | Human | Refugees | 6 | 1 |
|  | Drinking water | Refugees | 4 | 1 |
|  | Fomites | Refugees | 3 | 0 |
|  | Human | Host | 16 | 1 |
|  | Drinking water | Host | 3 | 0 |
|  | Fomites | Host | 3 | 0 |

**S3 Table.** Carbapenemase-producing *Escherichia coli* strains isolated from Lebanese patients in two major hospitals in proximity to the selected refugee camps in the same geographic area, collected concurrently with our community-based collection.

| **Sample ID** | **Hospital** | **Sample type** | **Carriage or infection** | **Year of isolation** | **Sex** | **Age (years)** |
| --- | --- | --- | --- | --- | --- | --- |
| O90C8 | Nini | Urine | Infection | 2021 | F | 81 |
| O90E9 | El Youssef | Urine | Infection | 2021 | M | 79 |
| O100H3 | Nini | Stool | Carriage | 2021 | M | 1 month* |
| O101A10 | Nini | Urine | Infection | 2022 | M | 5 |
| O100J3 | Nini | Urine | Infection | 2022 | F | 67 |
| O101B2 | Nini | Stool | Carriage | 2022 | M | 74 |
| O101D1 | Nini | Stool | Carriage | 2022 | M | 33 |
| O100G5 | Nini | Stool | Carriage | 2021 | M | 69 |
| O101B3 | Nini | Peritoneal fluid | Infection | 2022 | F | 7 |
| O101C2 | Nini | Urine | Infection | 2022 | F | 84 |
| O101A2 | Nini | Stool | Carriage | 2022 | M | 1 month* |
| O101D3 | Nini | Urine | Infection | 2022 | F | 86 |
| O89J5 | Nini | Axillary swab | Carriage | 2021 | M | 78 |
| O90A8 | Nini | Stool | Carriage | 2021 | M | 78 |
| O89J6 | Nini | Stool | Carriage | 2021 | M | 78 |
| O89I2 | Nini | Stool | Carriage | 2021 | M | 78 |
| O91C3 | Nini | Axillary swab | Carriage | 2021 | M | 78 |
| O100G9 | Nini | Urine | Infection | 2021 | M | 80 |
| O90D7 | Nini | Axillary swab | Carriage | 2021 | F | 65 |
| O100I10 | Nini | Stool | Carriage | 2021 | M | 78 |
| O91A5 | Nini | Stool | Carriage | 2021 | M | 40 |
| O90J2 | El Youssef | Urine | Infection | 2021 | F | 20 |
| O90A6 | El Youssef | Sputum | Infection | 2021 | M | 68 |
| O100F5 | Nini | Urine | Infection | 2021 | F | 1 |
| O91D1 | Nini | Urine | Infection | 2021 | F | 48 |
| O91D4 | Nini | Stool | Carriage | 2021 | F | 48 |
| O101E1 | El Youssef | Urine | Infection | 2021 | F | 81 |
| O101E5 | El Youssef | Urine | Infection | 2022 | F | 84 |
| O101I7 | Nini | Stool | Carriage | 2022 | M | 56 |
| O100J9 | Nini | Stool | Carriage | 2022 | M | 56 |
| O101C7 | Nini | Stool | Carriage | 2022 | M | 32 |

* Ages under 1 year are shown in months.

**S4 Table**. Accession numbers for Lebanese carbapenemase-producing *Escherichia coli* genomes analyzed in this study.

| **Genome ID** | **Genome Accession** | **Reference** |
| --- | --- | --- |
| O101F6 | SAMN47462387 | This study |
| O101F7 | SAMN47462388 | This study |
| O101G5 | SAMN47462389 | This study |
| O101G7 | SAMN47462390 | This study |
| O101G8 | SAMN47462391 | This study |
| O101H1 | SAMN47462392 | This study |
| O101H2 | SAMN47462393 | This study |
| O101H4 | SAMN47462394 | This study |
| O101H7 | SAMN47462395 | This study |
| O101H9 | SAMN47462396 | This study |
| O101I10 | SAMN47462398 | This study |
| O101J2 | SAMN47462399 | This study |
| O101J6 | SAMN47462400 | This study |
| O101H10 | SAMN47462397 | This study |
| O102A6 | SAMN47462402 | This study |
| O102A4 | SAMN47462401 | This study |
| O102C1 | SAMN47462403 | This study |
| O102C4 | SAMN47462404 | This study |
| O102D9 | SAMN47462405 | This study |
| O90C8 | SAMN47462406 | This study |
| O90E9 | SAMN47462407 | This study |
| O100H3 | SAMN47462408 | This study |
| O101A10 | SAMN47462409 | This study |
| O100J3 | SAMN47462410 | This study |
| O101B2 | SAMN47462411 | This study |
| O101D1 | SAMN47462412 | This study |
| O100G5 | SAMN47462413 | This study |
| O101B3 | SAMN47462414 | This study |
| O101C2 | SAMN47462415 | This study |
| O101D3 | SAMN47462417 | This study |
| O89J5 | SAMN47462418 | This study |
| O90A8 | SAMN47462419 | This study |
| O89J6 | SAMN47462420 | This study |
| O89I2 | SAMN47462421 | This study |
| O91C3 | SAMN47462422 | This study |
| O100G9 | SAMN47462423 | This study |
| O100I10 | SAMN47462425 | This study |
| O90D7 | SAMN47462424 | This study |
| O91A5 | SAMN47462426 | This study |
| O90J2 | SAMN47462427 | This study |
| O90A6 | SAMN47462428 | This study |
| O100F5 | SAMN47462429 | This study |
| O91D1 | SAMN47462430 | This study |
| O91D4 | SAMN47462431 | This study |
| O101E1 | SAMN47462432 | This study |
| O101E5 | SAMN47462433 | This study |
| O101I7 | SAMN47462434 | This study |
| O100J9 | SAMN47462435 | This study |
| O101C7 | SAMN47462436 | This study |
| O101A2 | SAMN47462416 | This study |
| ESC_KA0488AA | SAMN10058984 | Enterobase database |
| ESC_XA6565AA | SAMN18121011 | Enterobase database |
| ESC_XA6573AA | SAMN18120941 | Enterobase database |
| ESC_OB4326AA | SAMN44683812 | Enterobase database |
| ESC_OB4334AA | SAMN44683844 | Enterobase database |
| ESC_OB4340AA | SAMN44683838 | Enterobase database |
| ESC_OB4341AA | SAMN44683837 | Enterobase database |
| ESC_OB4347AA | SAMN44683832 | Enterobase database |
| ESC_OB4350AA | SAMN44683829 | Enterobase database |
| ESC_OB4351AA | SAMN44683828 | Enterobase database |
| ESC_OB4353AA | SAMN44683826 | Enterobase database |
| ESC_OB4355AA | SAMN44683825 | Enterobase database |
| ESC_OB4360AA | SAMN44683820 | Enterobase database |
| ESC_OB4364AA | SAMN44683816 | Enterobase database |
| ESC_OB4365AA | SAMN44683807 | Enterobase database |
| ESC_OB4366AA | SAMN44683806 | Enterobase database |
| ESC_OB4328AA | SAMN44683849 | Enterobase database |
| ESC_OB4330AA | SAMN44683847 | Enterobase database |
| ESC_OB4346AA | SAMN44683833 | Enterobase database |
| ESC_OB4352AA | SAMN44683827 | Enterobase database |
| ESC_OB4359AA | SAMN44683821 | Enterobase database |
| ESC_QB2479AA | SAMN46854017 | Enterobase database |
| ESC_QB2481AA | SAMN46854015 | Enterobase database |
| ESC_XA6634AA | SAMN18120948 | Enterobase database |
| ESC_YA7267AA | SAMN26208690 | Enterobase database |
| ESC_OB4332AA | SAMN44683810 | Enterobase database |
| ESC_QB2483AA | SAMN46854005 | Enterobase database |
| ESC_YA7266AA | SAMN26208691 | Enterobase database |
| ESC_QB2467AA | SAMN46854013 | Enterobase database |
| ESC_KA0489AA | SAMN10058992 | Enterobase database |
| 194 | SAMN44683806 | Enterobase database |
| 195 | SAMN44683807 | Enterobase database |
| 198 | SAMN44683810 | Enterobase database |
| 200 | SAMN44683812 | Enterobase database |
| 205 | SAMN44683816 | Enterobase database |
| 209 | SAMN44683820 | Enterobase database |
| 210 | SAMN44683821 | Enterobase database |
| 216 | SAMN44683825 | Enterobase database |
| 217 | SAMN44683826 | Enterobase database |
| 218 | SAMN44683827 | Enterobase database |
| 219 | SAMN44683828 | Enterobase database |
| 220 | SAMN44683829 | Enterobase database |
| 223 | SAMN44683832 | Enterobase database |
| 225 | SAMN44683833 | Enterobase database |
| 226 | SAMN44683834 | Enterobase database |
| 229 | SAMN44683837 | Enterobase database |
| 230 | SAMN44683838 | Enterobase database |
| 232 | SAMN44683840 | Enterobase database |
| 233 | SAMN44683841 | Enterobase database |
| 236 | SAMN44683844 | Enterobase database |
| 239 | SAMN44683847 | Enterobase database |
| 240 | SAMN44683848 | Enterobase database |
| 241 | SAMN44683849 | Enterobase database |
| AUH_IMP186 | SAMN06284153 | Enterobase database |
| AUH_IMP190 | SAMN06284178 | Enterobase database |
| AUH_IMP396 | SAMN06284481 | Enterobase database |
| 1856 | SAMN34104234 | NCBI |
| O85C4 | SAMN35126049 | https://doi.org/10.3389/fpubh.2023.1290912 |
| O85C6 | SAMN35126050 | https://doi.org/10.3389/fpubh.2023.1290913 |
| O85C10 | SAMN35126051 | https://doi.org/10.3389/fpubh.2023.1290914 |
| O85D6 | SAMN35126052 | https://doi.org/10.3389/fpubh.2023.1290915 |
| O85E2 | SAMN35126055 | https://doi.org/10.3389/fpubh.2023.1290916 |
| O85F3 | SAMN35126057 | https://doi.org/10.3389/fpubh.2023.1290917 |
| O85G1 | SAMN35126058 | https://doi.org/10.3389/fpubh.2023.1290918 |

**S5 Table**. Accession numbers of carbapenemase-producing Escherichia coli genomes from 2022 obtained via EnteroBase and analyzed in this study.

| **Genome ID** | **Genome Accession** | **Reference** |
| --- | --- | --- |
| ESC_CC1227AA_AS | SAMN25335192 | Enterobase database |
| ESC_CC2455AA_AS | SAMN25601421 | Enterobase database |
| ESC_CC3144AA_AS | SAMN25755511 | Enterobase database |
| ESC_CC4633AA_AS | SAMN26199862 | Enterobase database |
| ESC_CC4634AA_AS | SAMN26199860 | Enterobase database |
| ESC_CC8366AA_AS | SAMN26876063 | Enterobase database |
| ESC_CC8908AA_AS | SAMN27480191 | Enterobase database |
| ESC_CC8909AA_AS | SAMN27480222 | Enterobase database |
| ESC_CC8912AA_AS | SAMN27480200 | Enterobase database |
| ESC_CC9059AA_AS | SAMN27545061 | Enterobase database |
| ESC_CC9064AA_AS | SAMN27545042 | Enterobase database |
| ESC_CC9066AA_AS | SAMN27545036 | Enterobase database |
| ESC_CC9962AA_AS | Not available | Enterobase database |
| ESC_CC9756AA_AS | SAMN27631132 | Enterobase database |
| ESC_CC9856AA_AS | SAMN27568371 | Enterobase database |
| ESC_CC9857AA_AS | SAMN27568366 | Enterobase database |
| ESC_DC0051AA_AS | SAMN27739176 | Enterobase database |
| ESC_CC9964AA_AS | Not available | Enterobase database |
| ESC_DC0462AA_AS | SAMN27993071 | Enterobase database |
| ESC_DC0373AA_AS | SAMN27920055 | Enterobase database |
| ESC_DC0374AA_AS | SAMN27920054 | Enterobase database |
| ESC_DC0376AA_AS | SAMN27920059 | Enterobase database |
| ESC_DC2051AA_AS | SAMN28207093 | Enterobase database |
| ESC_DC2052AA_AS | SAMN28207092 | Enterobase database |
| ESC_DC2064AA_AS | SAMN28206112 | Enterobase database |
| ESC_DC2065AA_AS | SAMN28206114 | Enterobase database |
| ESC_DC2066AA_AS | SAMN28206113 | Enterobase database |
| ESC_DC2513AA_AS | SAMN28686320 | Enterobase database |
| ESC_DC2531AA_AS | SAMN28684433 | Enterobase database |
| ESC_DC2532AA_AS | SAMN28684428 | Enterobase database |
| ESC_DC2533AA_AS | SAMN28684423 | Enterobase database |
| ESC_DC2534AA_AS | SAMN28684422 | Enterobase database |
| ESC_DC2535AA_AS | SAMN28684421 | Enterobase database |
| ESC_DC2600AA_AS | SAMN28666826 | Enterobase database |
| ESC_DC2601AA_AS | SAMN28666831 | Enterobase database |
| ESC_DC2628AA_AS | SAMN28632846 | Enterobase database |
| ESC_DC2713AA_AS | SAMN28609094 | Enterobase database |
| ESC_DC2714AA_AS | SAMN28609093 | Enterobase database |
| ESC_DC2717AA_AS | SAMN28609089 | Enterobase database |
| ESC_DC2925AA_AS | SAMN28561901 | Enterobase database |
| ESC_DC2344AA_AS | SAMN28522342 | Enterobase database |
| ESC_DC3293AA_AS | SAMN28788571 | Enterobase database |
| ESC_DC3295AA_AS | SAMN28788532 | Enterobase database |
| ESC_DC4266AA_AS | SAMN28814731 | Enterobase database |
| ESC_DC4270AA_AS | SAMN28900513 | Enterobase database |
| ESC_DC4271AA_AS | SAMN28900512 | Enterobase database |
| ESC_DC4272AA_AS | SAMN28900520 | Enterobase database |
| ESC_DC5593AA_AS | SAMN28968557 | Enterobase database |
| ESC_DC5595AA_AS | SAMN28968554 | Enterobase database |
| ESC_DC7815AA_AS | SAMN26420027 | Enterobase database |
| ESC_DC7816AA_AS | SAMN26420026 | Enterobase database |
| ESC_DC9328AA_AS | SAMN29044366 | Enterobase database |
| ESC_EC0302AA_AS | SAMN29224576 | Enterobase database |
| ESC_EC0422AA_AS | SAMN29222408 | Enterobase database |
| ESC_EC0471AA_AS | Not available | Enterobase database |
| ESC_EC0881AA_AS | SAMN29719473 | Enterobase database |
| ESC_EC0957AA_AS | SAMN29633647 | Enterobase database |
| ESC_EC1824AA_AS | SAMN29578510 | Enterobase database |
| ESC_EC1834AA_AS | SAMN29577048 | Enterobase database |
| ESC_EC1835AA_AS | SAMN29577047 | Enterobase database |
| ESC_EC1908AA_AS | SAMN29507288 | Enterobase database |
| ESC_EC1934AA_AS | SAMN29507031 | Enterobase database |
| ESC_EC1943AA_AS | SAMN29506980 | Enterobase database |
| ESC_EC1980AA_AS | SAMN29506634 | Enterobase database |
| ESC_EC2082AA_AS | SAMN29473047 | Enterobase database |
| ESC_EC2123AA_AS | SAMN29440606 | Enterobase database |
| ESC_EC3762AA_AS | SAMN29868717 | Enterobase database |
| ESC_FC1983AA_AS | SAMN29894093 | Enterobase database |
| ESC_EC3832AA_AS | SAMN29894085 | Enterobase database |
| ESC_EC3833AA_AS | SAMN29894078 | Enterobase database |
| ESC_EC3834AA_AS | SAMN29894075 | Enterobase database |
| ESC_FC4333AA_AS | SAMN30050890 | Enterobase database |
| ESC_FC4334AA_AS | SAMN30127328 | Enterobase database |
| ESC_FC4340AA_AS | SAMN30109953 | Enterobase database |
| ESC_FC4374AA_AS | SAMN30104316 | Enterobase database |
| ESC_FC4376AA_AS | SAMN29614905 | Enterobase database |
| ESC_FC4397AA_AS | SAMN29475785 | Enterobase database |
| ESC_FC4402AA_AS | SAMN29475780 | Enterobase database |
| ESC_FC4404AA_AS | SAMN29475778 | Enterobase database |
| ESC_FC4407AA_AS | SAMN29475773 | Enterobase database |
| ESC_FC4410AA_AS | SAMN29475770 | Enterobase database |
| ESC_FC4411AA_AS | SAMN29475769 | Enterobase database |
| ESC_FC4412AA_AS | SAMN29475768 | Enterobase database |
| ESC_FC4413AA_AS | SAMN29475767 | Enterobase database |
| ESC_FC4414AA_AS | SAMN29475766 | Enterobase database |
| ESC_FC4415AA_AS | SAMN29475759 | Enterobase database |
| ESC_FC4416AA_AS | SAMN29475758 | Enterobase database |
| ESC_FC4417AA_AS | SAMN29475757 | Enterobase database |
| ESC_FC4418AA_AS | SAMN29475756 | Enterobase database |
| ESC_FC4419AA_AS | SAMN29475755 | Enterobase database |
| ESC_FC4420AA_AS | SAMN29475754 | Enterobase database |
| ESC_FC4421AA_AS | SAMN29475753 | Enterobase database |
| ESC_FC4422AA_AS | SAMN29475752 | Enterobase database |
| ESC_FC4423AA_AS | SAMN29475751 | Enterobase database |
| ESC_FC4424AA_AS | SAMN29475750 | Enterobase database |
| ESC_FC4425AA_AS | SAMN29475748 | Enterobase database |
| ESC_FC4426AA_AS | SAMN29475747 | Enterobase database |
| ESC_FC4428AA_AS | SAMN29475745 | Enterobase database |
| ESC_FC4429AA_AS | SAMN29475744 | Enterobase database |
| ESC_FC4430AA_AS | SAMN29475743 | Enterobase database |
| ESC_FC4431AA_AS | SAMN29475724 | Enterobase database |
| ESC_EC6874AA_AS | SAMN30031202 | Enterobase database |
| ESC_EC6875AA_AS | SAMN30031130 | Enterobase database |
| ESC_EC8472AA_AS | SAMN30370664 | Enterobase database |
| ESC_FC6191AA_AS | SAMN30415608 | Enterobase database |
| ESC_FC1600AA_AS | SAMN30429957 | Enterobase database |
| ESC_EC9106AA_AS | Not available | Enterobase database |
| ESC_FC7669AA_AS | SAMN30497978 | Enterobase database |
| ESC_FC7671AA_AS | SAMN30497938 | Enterobase database |
| ESC_GC7538AA_AS | SAMN30551355 | Enterobase database |
| ESC_FC7768AA_AS | SAMN30551353 | Enterobase database |
| ESC_FC1958AA_AS | SAMN30556253 | Enterobase database |
| ESC_FC1960AA_AS | SAMN30556250 | Enterobase database |
| ESC_FC1968AA_AS | Not available | Enterobase database |
| ESC_FC8411AA_AS | SAMN30595837 | Enterobase database |
| ESC_FC3328AA_AS | SAMN30655613 | Enterobase database |
| ESC_FC3329AA_AS | SAMN30655643 | Enterobase database |
| ESC_FC3333AA_AS | SAMN30651439 | Enterobase database |
| ESC_FC3470AA_AS | Not available | Enterobase database |
| ESC_FC3894AA_AS | SAMN30617503 | Enterobase database |
| ESC_FC3991AA_AS | SAMN30811842 | Enterobase database |
| ESC_FC4167AA_AS | SAMN30863456 | Enterobase database |
| ESC_FC4466AA_AS | SAMN30874014 | Enterobase database |
| ESC_FC4563AA_AS | SAMN30889564 | Enterobase database |
| ESC_FC4565AA_AS | SAMN30889313 | Enterobase database |
| ESC_FC4566AA_AS | SAMN30889312 | Enterobase database |
| ESC_GC3839AA_AS | SAMN30889238 | Enterobase database |
| ESC_FC4570AA_AS | SAMN30889237 | Enterobase database |
| ESC_FC4571AA_AS | SAMN30889250 | Enterobase database |
| ESC_FC5302AA_AS | SAMN30963922 | Enterobase database |
| ESC_FC5634AA_AS | SAMN31037988 | Enterobase database |
| ESC_FC5636AA_AS | SAMN31037983 | Enterobase database |
| ESC_FC5637AA_AS | SAMN31037979 | Enterobase database |
| ESC_FC5639AA_AS | SAMN31037972 | Enterobase database |
| ESC_FC5640AA_AS | SAMN31037957 | Enterobase database |
| ESC_GC7107AA_AS | SAMN31152654 | Enterobase database |
| ESC_FC6724AA_AS | SAMN31151752 | Enterobase database |
| ESC_FC7904AA_AS | SAMN31230816 | Enterobase database |
| ESC_FC7905AA_AS | SAMN31230813 | Enterobase database |
| ESC_GC0023AA_AS | SAMN31208384 | Enterobase database |
| ESC_GC0036AA_AS | SAMN31261076 | Enterobase database |
| ESC_GC0059AA_AS | SAMN31249867 | Enterobase database |
| ESC_GC0060AA_AS | SAMN31249865 | Enterobase database |
| ESC_GC0061AA_AS | SAMN31249858 | Enterobase database |
| ESC_GC0062AA_AS | SAMN31249855 | Enterobase database |
| ESC_GC0290AA_AS | SAMN31275289 | Enterobase database |
| ESC_GC0552AA_AS | SAMN31186970 | Enterobase database |
| ESC_GC0829AA_AS | SAMN31282212 | Enterobase database |
| ESC_GC1212AA_AS | SAMN31309204 | Enterobase database |
| ESC_GC2266AA_AS | SAMN31384132 | Enterobase database |
| ESC_GC2269AA_AS | SAMN31384003 | Enterobase database |
| ESC_GC2299AA_AS | SAMN31390131 | Enterobase database |
| ESC_GC2303AA_AS | SAMN31388771 | Enterobase database |
| ESC_GC2304AA_AS | SAMN31388766 | Enterobase database |
| ESC_GC2334AA_AS | SAMN31390546 | Enterobase database |
| ESC_GC2371AA_AS | SAMN31399571 | Enterobase database |
| ESC_GC2475AA_AS | SAMN31400868 | Enterobase database |
| ESC_GC3067AA_AS | SAMN31419960 | Enterobase database |
| ESC_GC3677AA_AS | SAMN31438045 | Enterobase database |
| ESC_GC3766AA_AS | Not available | Enterobase database |
| ESC_GC3775AA_AS | SAMN31460738 | Enterobase database |
| ESC_GC3776AA_AS | SAMN31460743 | Enterobase database |
| ESC_GC3777AA_AS | SAMN31460742 | Enterobase database |
| ESC_GC3778AA_AS | SAMN31460732 | Enterobase database |
| ESC_GC3840AA_AS | Not available | Enterobase database |
| ESC_GC4008AA_AS | SAMN31488179 | Enterobase database |
| ESC_GC4013AA_AS | SAMN31488183 | Enterobase database |
| ESC_GC4014AA_AS | SAMN31488182 | Enterobase database |
| ESC_GC4015AA_AS | SAMN31488181 | Enterobase database |
| ESC_GC4017AA_AS | SAMN31484131 | Enterobase database |
| ESC_GC5588AA_AS | SAMN31571012 | Enterobase database |
| ESC_GC5590AA_AS | SAMN31571006 | Enterobase database |
| ESC_GC6469AA_AS | SAMN31611583 | Enterobase database |
| ESC_GC6810AA_AS | SAMN29755197 | Enterobase database |
| ESC_GC6816AA_AS | SAMN29755174 | Enterobase database |
| ESC_GC6881AA_AS | SAMN31684555 | Enterobase database |
| ESC_GC6882AA_AS | SAMN31684552 | Enterobase database |
| ESC_GC7132AA_AS | SAMN31715202 | Enterobase database |
| ESC_GC7133AA_AS | SAMN31715211 | Enterobase database |
| ESC_GC7134AA_AS | SAMN31715210 | Enterobase database |
| ESC_GC7175AA_AS | SAMN31711875 | Enterobase database |
| ESC_GC7337AA_AS | SAMN31740958 | Enterobase database |
| ESC_GC7338AA_AS | SAMN31740955 | Enterobase database |
| ESC_GC7399AA_AS | SAMN31759542 | Enterobase database |
| ESC_GC7414AA_AS | SAMN31774413 | Enterobase database |
| ESC_GC7506AA_AS | SAMN31779636 | Enterobase database |
| ESC_GC8440AA_AS | SAMN31819813 | Enterobase database |
| ESC_GC8441AA_AS | SAMN31819823 | Enterobase database |
| ESC_GC8442AA_AS | SAMN31819822 | Enterobase database |
| ESC_GC8471AA_AS | SAMN31854200 | Enterobase database |
| ESC_GC8472AA_AS | SAMN31854212 | Enterobase database |
| ESC_GC9784AA_AS | SAMN31951789 | Enterobase database |
| ESC_GC9785AA_AS | SAMN31951799 | Enterobase database |
| ESC_GC9787AA_AS | SAMN32011618 | Enterobase database |
| ESC_HC0400AA_AS | SAMN32095245 | Enterobase database |
| ESC_HC0642AA_AS | SAMN32130570 | Enterobase database |
| ESC_HC0643AA_AS | SAMN32130569 | Enterobase database |
| ESC_HC0644AA_AS | SAMN32130568 | Enterobase database |
| ESC_HC0675AA_AS | SAMN32116101 | Enterobase database |
| ESC_HC3086AA_AS | SAMN32244630 | Enterobase database |
| ESC_HC3087AA_AS | SAMN32244629 | Enterobase database |
| ESC_HC4467AA_AS | SAMN32192645 | Enterobase database |
| ESC_HC4666AA_AS | SAMN32314467 | Enterobase database |
| ESC_HC4685AA_AS | SAMEA112251228 | Enterobase database |
| ESC_HC4699AA_AS | SAMN32372894 | Enterobase database |
| ESC_HC4700AA_AS | SAMN32372891 | Enterobase database |
| ESC_HC5412AA_AS | SAMN32470444 | Enterobase database |
| ESC_HC5413AA_AS | SAMN32470428 | Enterobase database |
| ESC_HC7060AA_AS | SAMN32646359 | Enterobase database |
| ESC_HC7810AA_AS | Not available | Enterobase database |
| ESC_HC7933AA_AS | SAMN32733995 | Enterobase database |
| ESC_HC7934AA_AS | SAMN32733968 | Enterobase database |
| ESC_HC8093AA_AS | SAMN32768121 | Enterobase database |
| ESC_HC8307AA_AS | SAMN32801833 | Enterobase database |
| ESC_HC8308AA_AS | SAMN32801840 | Enterobase database |
| ESC_HC8309AA_AS | SAMN32801838 | Enterobase database |
| ESC_HC8313AA_AS | SAMN32801548 | Enterobase database |
| ESC_HC8345AA_AS | SAMN32801356 | Enterobase database |
| ESC_HC8466AA_AS | SAMN32816952 | Enterobase database |
| ESC_HC8469AA_AS | SAMN32816991 | Enterobase database |
| ESC_HC8470AA_AS | SAMN32816987 | Enterobase database |
| ESC_HC8471AA_AS | SAMN32816981 | Enterobase database |
| ESC_HC8472AA_AS | SAMN32816977 | Enterobase database |
| ESC_HC8473AA_AS | SAMN32816974 | Enterobase database |
| ESC_HC8474AA_AS | SAMN32816968 | Enterobase database |
| ESC_HC8476AA_AS | SAMN32816963 | Enterobase database |
| ESC_HC8697AA_AS | Not available | Enterobase database |
| ESC_HC8699AA_AS | Not available | Enterobase database |
| ESC_HC8986AA_AS | SAMN32908209 | Enterobase database |
| ESC_HC8987AA_AS | SAMN32908208 | Enterobase database |
| ESC_HC9030AA_AS | SAMN32942628 | Enterobase database |
| ESC_HC9436AA_AS | SAMN33005080 | Enterobase database |
| ESC_HC9517AA_AS | SAMN33044217 | Enterobase database |
| ESC_HC9954AA_AS | SAMN33190919 | Enterobase database |
| ESC_HC9957AA_AS | SAMN33190910 | Enterobase database |
| ESC_IC0194AA_AS | SAMN33228744 | Enterobase database |
| ESC_IC0196AA_AS | Not available | Enterobase database |
| ESC_IC0653AA_AS | SAMN33285646 | Enterobase database |
| ESC_IC0654AA_AS | SAMN33285655 | Enterobase database |
| ESC_IC0655AA_AS | SAMN33285620 | Enterobase database |
| ESC_IC0677AA_AS | SAMN33304692 | Enterobase database |
| ESC_IC0680AA_AS | SAMN33304683 | Enterobase database |
| ESC_IC1800AA_AS | SAMN33314763 | Enterobase database |
| ESC_IC2752AA_AS | SAMN33193799 | Enterobase database |
| ESC_IC2874AA_AS | Not available | Enterobase database |
| ESC_IC3266AA_AS | SAMN33611478 | Enterobase database |
| ESC_IC3268AA_AS | SAMN33611466 | Enterobase database |
| ESC_IC3269AA_AS | SAMN33611467 | Enterobase database |
| ESC_IC3271AA_AS | SAMN33611463 | Enterobase database |
| ESC_IC4213AA_AS | SAMN33797363 | Enterobase database |
| ESC_IC4214AA_AS | SAMN33797361 | Enterobase database |
| ESC_IC4638AA_AS | SAMN33830160 | Enterobase database |
| ESC_IC5337AA_AS | SAMN33845280 | Enterobase database |
| ESC_IC5338AA_AS | SAMN33845271 | Enterobase database |
| ESC_IC5926AA_AS | Not available | Enterobase database |
| ESC_IC7161AA_AS | SAMN34030548 | Enterobase database |
| ESC_IC7162AA_AS | SAMN34030554 | Enterobase database |
| ESC_IC7342AA_AS | SAMN34104097 | Enterobase database |
| ESC_IC8488AA_AS | SAMN34174871 | Enterobase database |
| ESC_IC8612AA_AS | SAMN34191137 | Enterobase database |
| ESC_IC8621AA_AS | SAMN34190924 | Enterobase database |
| ESC_IC8900AA_AS | SAMN34211735 | Enterobase database |
| ESC_IC9473AA_AS | SAMN33797271 | Enterobase database |
| ESC_IC9500AA_AS | SAMN33797195 | Enterobase database |
| ESC_IC9709AA_AS | SAMN34239450 | Enterobase database |
| ESC_IC9711AA_AS | SAMN34239448 | Enterobase database |
| ESC_IC9713AA_AS | SAMN34239443 | Enterobase database |
| ESC_JC0196AA_AS | SAMN34251416 | Enterobase database |
| ESC_JC0199AA_AS | SAMN34250961 | Enterobase database |
| ESC_JC1061AA_AS | SAMN34236057 | Enterobase database |
| ESC_JC4581AA_AS | SAMN34411575 | Enterobase database |
| ESC_JC4602AA_AS | SAMN34411512 | Enterobase database |
| ESC_JC7582AA_AS | SAMN35130653 | Enterobase database |
| ESC_JC7617AA_AS | SAMN35133125 | Enterobase database |
| ESC_JC7618AA_AS | SAMN35133124 | Enterobase database |
| ESC_JC8156AA_AS | SAMN35440261 | Enterobase database |
| ESC_JC8157AA_AS | SAMN35440259 | Enterobase database |
| ESC_JC8159AA_AS | SAMN35440275 | Enterobase database |
| ESC_JC8163AA_AS | SAMN35440267 | Enterobase database |
| ESC_JC8166AA_AS | SAMN35440193 | Enterobase database |
| ESC_JC9021AA_AS | SAMN29866075 | Enterobase database |
| ESC_JC9219AA_AS | SAMN35573237 | Enterobase database |
| ESC_JC9416AA_AS | SAMN35573225 | Enterobase database |
| ESC_JC9723AA_AS | SAMN35573236 | Enterobase database |
| ESC_JC9724AA_AS | SAMN35573229 | Enterobase database |
| ESC_JC9725AA_AS | SAMN35573230 | Enterobase database |
| ESC_JC9811AA_AS | SAMN35573233 | Enterobase database |
| ESC_JC9812AA_AS | SAMN35573231 | Enterobase database |
| ESC_JC9814AA_AS | SAMN35573224 | Enterobase database |
| ESC_KC1779AA_AS | SAMN35735605 | Enterobase database |
| ESC_KC1782AA_AS | SAMN35735642 | Enterobase database |
| ESC_KC1784AA_AS | SAMN35735640 | Enterobase database |
| ESC_KC1785AA_AS | SAMN35735633 | Enterobase database |
| ESC_KC1786AA_AS | SAMN35735629 | Enterobase database |
| ESC_KC1787AA_AS | SAMN35735601 | Enterobase database |
| ESC_KC1788AA_AS | SAMN35735627 | Enterobase database |
| ESC_KC1789AA_AS | SAMN35735626 | Enterobase database |
| ESC_KC1790AA_AS | SAMN35735622 | Enterobase database |
| ESC_KC1791AA_AS | SAMN35735600 | Enterobase database |
| ESC_KC1792AA_AS | SAMN35735612 | Enterobase database |
| ESC_KC1841AA_AS | SAMN35841712 | Enterobase database |
| ESC_KC3359AA_AS | SAMN36340822 | Enterobase database |
| ESC_KC3361AA_AS | SAMN36340820 | Enterobase database |
| ESC_KC3364AA_AS | SAMN36340817 | Enterobase database |
| ESC_KC3367AA_AS | SAMN36340814 | Enterobase database |
| ESC_KC3369AA_AS | SAMN36340812 | Enterobase database |
| ESC_KC3370AA_AS | SAMN36340811 | Enterobase database |
| ESC_KC4860AA_AS | SAMN36496003 | Enterobase database |
| ESC_LC4114AA_AS | SAMEA112938252 | Enterobase database |
| ESC_LC4436AA_AS | SAMN37305322 | Enterobase database |
| ESC_LC7969AA_AS | SAMEA114236657 | Enterobase database |
| ESC_LC7973AA_AS | SAMEA114236658 | Enterobase database |
| ESC_MC3665AA_AS | SAMN38223595 | Enterobase database |
| ESC_MC3667AA_AS | SAMN38223587 | Enterobase database |
| ESC_MC4583AA_AS | SAMN38336107 | Enterobase database |
| ESC_MC6531AA_AS | SAMN38765945 | Enterobase database |
| ESC_MC9015AA_AS | Not available | Enterobase database |
| ESC_NC0023AA_AS | SAMN39403978 | Enterobase database |
| ESC_NC1405AA_AS | Not available | Enterobase database |
| ESC_NC8731AA_AS | SAMN39782946 | Enterobase database |
| ESC_NC4029AA_AS | SAMN39950455 | Enterobase database |
| ESC_NC4030AA_AS | SAMN39950427 | Enterobase database |
| ESC_NC4061AA_AS | SAMN39950440 | Enterobase database |
| ESC_NC4063AA_AS | SAMN39950439 | Enterobase database |
| ESC_NC4085AA_AS | SAMN39950426 | Enterobase database |
| ESC_NC5936AA_AS | SAMN39402434 | Enterobase database |
| ESC_NC7071AA_AS | Not available | Enterobase database |
| ESC_NC7793AA_AS | SAMN40354501 | Enterobase database |
| ESC_NC7816AA_AS | Not available | Enterobase database |
| ESC_OC4148AA_AS | SAMN40992285 | Enterobase database |
| ESC_OC4620AA_AS | SAMN41064267 | Enterobase database |
| ESC_OC5426AA_AS | SAMN35719123 | Enterobase database |
| ESC_PC0318AA_AS | SAMN41499999 | Enterobase database |
| ESC_PC0327AA_AS | SAMN41500016 | Enterobase database |
| ESC_PC0328AA_AS | SAMN41500015 | Enterobase database |
| ESC_PC0329AA_AS | SAMN41500014 | Enterobase database |
| ESC_PC0330AA_AS | SAMN41500013 | Enterobase database |
| ESC_PC0331AA_AS | SAMN41500012 | Enterobase database |
| ESC_PC0332AA_AS | SAMN41500011 | Enterobase database |
| ESC_PC0333AA_AS | SAMN41500010 | Enterobase database |
| ESC_PC0334AA_AS | SAMN41500009 | Enterobase database |
| ESC_PC0335AA_AS | SAMN41499996 | Enterobase database |
| ESC_PC0336AA_AS | SAMN41500005 | Enterobase database |
| ESC_PC0343AA_AS | SAMN41500034 | Enterobase database |
| ESC_PC0344AA_AS | SAMN41500033 | Enterobase database |
| ESC_PC0345AA_AS | SAMN41500032 | Enterobase database |
| ESC_PC0346AA_AS | SAMN41500031 | Enterobase database |
| ESC_PC0347AA_AS | SAMN41500030 | Enterobase database |
| ESC_PC0348AA_AS | SAMN41500029 | Enterobase database |
| ESC_PC0349AA_AS | SAMN41499998 | Enterobase database |
| ESC_PC0350AA_AS | SAMN41500028 | Enterobase database |
| ESC_PC0351AA_AS | SAMN41500027 | Enterobase database |
| ESC_PC0353AA_AS | SAMN41500025 | Enterobase database |
| ESC_PC0354AA_AS | SAMN41500024 | Enterobase database |
| ESC_PC0355AA_AS | SAMN41500023 | Enterobase database |
| ESC_PC0356AA_AS | SAMN41500022 | Enterobase database |
| ESC_PC0357AA_AS | SAMN41500021 | Enterobase database |
| ESC_PC0358AA_AS | SAMN41499997 | Enterobase database |
| ESC_PC0359AA_AS | SAMN41500020 | Enterobase database |
| ESC_PC0360AA_AS | SAMN41500019 | Enterobase database |
| ESC_PC0361AA_AS | SAMN41500018 | Enterobase database |
| ESC_PC0362AA_AS | SAMN41500017 | Enterobase database |
| ESC_PC0363AA_AS | SAMN41500007 | Enterobase database |
| ESC_PC0364AA_AS | SAMN41500002 | Enterobase database |
| ESC_PC0365AA_AS | SAMN41500001 | Enterobase database |
| ESC_PC0366AA_AS | SAMN41500006 | Enterobase database |
| ESC_PC0367AA_AS | SAMN41500000 | Enterobase database |
| ESC_PC0393AA_AS | SAMN41500008 | Enterobase database |
| ESC_PC0394AA_AS | SAMN41500003 | Enterobase database |
| ESC_PC0396AA_AS | SAMN41499995 | Enterobase database |
| ESC_PC0397AA_AS | SAMN41500163 | Enterobase database |
| ESC_PC2414AA_AS | SAMN41581443 | Enterobase database |
| ESC_PC5905AA_AS | SAMN41841674 | Enterobase database |
| ESC_PC5906AA_AS | SAMN41841673 | Enterobase database |
| ESC_PC5938AA_AS | SAMN41844158 | Enterobase database |
| ESC_PC5939AA_AS | SAMN41844157 | Enterobase database |
| ESC_PC5940AA_AS | SAMN41844156 | Enterobase database |
| ESC_PC9630AA_AS | SAMN42382070 | Enterobase database |
| ESC_PC9627AA_AS | SAMN42382053 | Enterobase database |
| ESC_RC8167AA_AS | SAMEA112330465 | Enterobase database |
| ESC_RC8191AA_AS | SAMEA112330353 | Enterobase database |
| ESC_RC8224AA_AS | SAMEA112330386 | Enterobase database |
| ESC_RC6034AA_AS | SAMEA113604662 | Enterobase database |
| ESC_SC2197AA_AS | SAMN41098724 | Enterobase database |
| ESC_UC0941AA_AS | SAMN44683812 | Enterobase database |
| ESC_UC0943AA_AS | SAMN44683849 | Enterobase database |
| ESC_UC0944AA_AS | SAMN44683848 | Enterobase database |
| ESC_UC0945AA_AS | SAMN44683847 | Enterobase database |
| ESC_UC0947AA_AS | SAMN44683810 | Enterobase database |
| ESC_UC0949AA_AS | SAMN44683844 | Enterobase database |
| ESC_UC0952AA_AS | SAMN44683841 | Enterobase database |
| ESC_UC0955AA_AS | SAMN44683838 | Enterobase database |
| ESC_UC0956AA_AS | SAMN44683837 | Enterobase database |
| ESC_UC0961AA_AS | SAMN44683833 | Enterobase database |
| ESC_UC0962AA_AS | SAMN44683832 | Enterobase database |
| ESC_UC0965AA_AS | SAMN44683829 | Enterobase database |
| ESC_UC0966AA_AS | SAMN44683828 | Enterobase database |
| ESC_UC0967AA_AS | SAMN44683827 | Enterobase database |
| ESC_UC0968AA_AS | SAMN44683826 | Enterobase database |
| ESC_UC0970AA_AS | SAMN44683825 | Enterobase database |
| ESC_UC0974AA_AS | SAMN44683821 | Enterobase database |
| ESC_UC0975AA_AS | SAMN44683820 | Enterobase database |
| ESC_UC0979AA_AS | SAMN44683816 | Enterobase database |
| ESC_UC0980AA_AS | SAMN44683807 | Enterobase database |
| ESC_UC0981AA_AS | SAMN44683806 | Enterobase database |
| ESC_WC1707AA_AS | SAMEA117590635 | Enterobase database |
| ESC_VC9606AA_AS | Not available | Enterobase database |
| ESC_VC9686AA_AS | Not available | Enterobase database |
| ESC_WC0104AA_AS | SAMN46828803 | Enterobase database |
| ESC_WC0105AA_AS | SAMN46828802 | Enterobase database |
| ESC_WC0455AA_AS | Not available | Enterobase database |
| ESC_WC0456AA_AS | Not available | Enterobase database |
| ESC_WC0457AA_AS | Not available | Enterobase database |
| ESC_WC0458AA_AS | SAMN47462400 | Enterobase database |
| ESC_WC0459AA_AS | SAMN47462399 | Enterobase database |
| ESC_WC0460AA_AS | Not available | Enterobase database |
| ESC_WC0461AA_AS | SAMN47462389 | Enterobase database |
| ESC_WC8412AA_AS | SAMEA117735032 | Enterobase database |
| ESC_WC1412AA_AS | SAMN47462396 | Enterobase database |
| ESC_WC1414AA_AS | SAMN47462398 | Enterobase database |
| ESC_WC1415AA_AS | SAMN47462395 | Enterobase database |
| ESC_WC1416AA_AS | SAMN47462402 | Enterobase database |
| ESC_WC1421AA_AS | SAMN47462394 | Enterobase database |
| ESC_WC1422AA_AS | SAMN47462403 | Enterobase database |
| ESC_WC1423AA_AS | SAMN47462388 | Enterobase database |
| ESC_WC1424AA_AS | SAMN47462397 | Enterobase database |
| ESC_WC1425AA_AS | SAMN47462392 | Enterobase database |
| ESC_WC1426AA_AS | SAMN47462405 | Enterobase database |
| ESC_WC1427AA_AS | SAMN47462404 | Enterobase database |
| ESC_WC1429AA_AS | SAMN47462390 | Enterobase database |
| ESC_WC1430AA_AS | Not available | Enterobase database |
| ESC_WC1431AA_AS | Not available | Enterobase database |
| ESC_WC1432AA_AS | SAMN47462401 | Enterobase database |
| ESC_WC1654AA_AS | SAMN47462393 | Enterobase database |
| ESC_WC1655AA_AS | SAMN47462387 | Enterobase database |
| ESC_WC1657AA_AS | Not available | Enterobase database |
| ESC_WC1658AA_AS | Not available | Enterobase database |
| ESC_WC1659AA_AS | Not available | Enterobase database |
| ESC_WC1674AA_AS | Not available | Enterobase database |
| ESC_WC1675AA_AS | Not available | Enterobase database |
| ESC_WC1676AA_AS | Not available | Enterobase database |
| ESC_WC5110AA_AS | Not available | Enterobase database |
| ESC_WC5179AA_AS | Not available | Enterobase database |
| ESC_WC5212AA_AS | Not available | Enterobase database |
| ESC_WC5286AA_AS | Not available | Enterobase database |
| ESC_WC5214AA_AS | Not available | Enterobase database |
| ESC_WC5216AA_AS | Not available | Enterobase database |
| ESC_WC5219AA_AS | Not available | Enterobase database |
| ESC_WC5235AA_AS | Not available | Enterobase database |
| ESC_WC5236AA_AS | Not available | Enterobase database |
| ESC_WC5331AA_AS | Not available | Enterobase database |
| ESC_WC5357AA_AS | Not available | Enterobase database |
| ESC_WC5358AA_AS | Not available | Enterobase database |
| ESC_WC5362AA_AS | Not available | Enterobase database |
| ESC_WC5363AA_AS | Not available | Enterobase database |
| ESC_WC5364AA_AS | Not available | Enterobase database |
| ESC_WC5367AA_AS | Not available | Enterobase database |
| ESC_XC7052AA_AS | SAMEA117683899 | Enterobase database |
| ESC_XC7069AA_AS | SAMEA117683897 | Enterobase database |
| ESC_WC9960AA_AS | SAMN47601721 | Enterobase database |
| ESC_XC1141AA_AS | SAMN46558897 | Enterobase database |
| ESC_YC4478AA_AS | SAMEA117624492 | Enterobase database |
| ESC_YC4487AA_AS | SAMEA117624493 | Enterobase database |
| ESC_XC8592AA_AS | SAMN48581747 | Enterobase database |
| ESC_XC9681AA_AS | SAMN48723294 | Enterobase database |
| ESC_ZC5449AA_AS | SAMN50601325 | Enterobase database |

**S7 Table.** Prevalence of antimicrobial resistance to clinically and veterinary relevant antibiotics in community (n=19) and clinical (n=31) carbapenemase-producing *Escherichia coli* isolates recovered in this study.

| **Antimicrobial agents** |  |  |  | **Total**  **n=50** | **Community (human, animal, or environment)**  **n=19** | **Community (human)**  **n=10** | **Community (animal or environment)**  **n=9** | **Hospital (human)**  **n=31** |
| --- | --- | --- | --- | --- | --- | --- | --- | --- |
|  |  |  |  |  |  |  |  |  |
|  | **MIC Range** | **MIC_50_** | **MIC_90_** | **R (%)** | **R (%)** | **R (%)** | **R (%)** | **R (%)** |
| **Mecillinam** | ≤2..>16 | 8 | >16 | 60 | 58 | 60 | 56 | 61 |
| **Cefoxitin** | ≤4..>32 | >32 | >32 | 94 | 84 | 80 | 89 | 100 |
| **Cefotaxime** | ≤0.5..>8 | >8 | >8 | 98 | 95 | 100 | 89 | 100 |
| **Ceftazidime** | ≤0.25..>32 | >32 | >32 | 96 | 90 | 90 | 89 | 100 |
| **Ceftazidime- avibactam** | ≤0.25..>32 | >32 | >32 | 82 | 84 | 80 | 89 | 81 |
| **Cephalexin** | ≤4..128 | >128 | >128 | 98 | 95 | 100 | 89 | 100 |
| **Aztreonam** | ≤0.06..>16 | >16 | >16 | 92 | 95 | 100 | 89 | 90 |
| **Aztreonam-avibactam** | ≤0.03..>17 | 1 | 4 | 10 | 0 | 0 | 0 | 16 |
| **Cefepime** | ≤0.5..>18 | >16 | >16 | 96 | 90 | 90 | 89 | 100 |
| **Ceftolozane- tazobactam** | 0.5..>32 | >8 | >16 | 96 | 90 | 90 | 89 | 100 |
| **Cefiderocol** | 0.5..>32 | 2 | 8 | 38 | 37 | 40 | 33 | 39 |
| **Ertapenem*** | 0.5..>8 | 8 | >8 | 98 | 95 | 90 | 100 | 100 |
| **Imipenem** | 0.25..32 | 4 | 16 | 82 | 84 | 80 | 89 | 81 |
| **Imipenem-relebactam** | 0.25..>16 | 4 | 16 | 84 | 84 | 80 | 89 | 84 |
| **Meropenem** | 0.12..>32 | 4 | >32 | 88 | 90 | 100 | 89 | 87 |
| **Meropenem- vaborbactam^!^** | 0.12..>32 | 4 | 32 | 74 | 79 | 90 | 63 | 71 |
| **Levofloxacin** | 0.25..>4 | >4 | >4 | 96 | 90 | 90 | 89 | 100 |
| **Ciprofloxacin** | 0.12..>4 | >2 | >2 | 98 | 95 | 100 | 89 | 100 |
| **Delafloxacin** | 0.06..>2 | 2 | >2 | 98 | 95 | 100 | 89 | 100 |
| **Amikacin** | ≤4..>32 | ≤4 | ≤5 | 6 | 0 | 0 | 0 | 10 |
| **Gentamicin** | ≤1..>8 | ≤1 | >8 | 32 | 16 | 20 | 11 | 42 |
| **Tobramycin** | ≤1..>8 | ≤1 | >8 | 38 | 16 | 20 | 11 | 52 |
| **Streptomycin*** | ≤4..>32 | 8 | 32 | 56 | 63 | 70 | 56 | 52 |
| **Apramycin*** | ≤0.5..16 | 2 | 4 | 0 | 0 | 0 | 0 | 0 |
| **Neomycin*** | ≤4 | ≤4 | ≤4 | 0 | 0 | 0 | 0 | 0 |
| **Tetracycline*** | ≤2..>16 | 16 | >16 | 76 | 79 | 70 | 89 | 74 |
| **Tigecycline** | ≤0.25..1 | ≤0.25 | 0.5 | 2 | 0 | 0 | 0 | 3 |
| **Eravacycline** | 0.03..1 | 0.12 | 0.5 | 2 | 0 | 0 | 0 | 3 |
| **Chloramphenicol** | ≤4..>16 | ≤4 | >16 | 36 | 21 | 30 | 0 | 45 |
| **Florfenicol*** | ≤2..32 | 8 | 16 | 36 | 37 | 50 | 0 | 35 |
| **Colistin** | ≤0.5..2 | ≤0.5 | 0.5 | 0 | 0 | 0 | 0 | 0 |
| **Fosfomycin** | ≤4..>32 | ≤4 | ≤4 | 4 | 0 | 0 | 0 | 6 |
| **Nitrofurantoin** | ≤32..128 | ≤32 | ≤32 | 4 | 0 | 0 | 0 | 6 |
| **Trimethoprim** | ≤1..>8 | >8 | >8 | 76 | 63 | 70 | 56 | 84 |
| **Sulfamethoxazole*** | ≤32..>512 | >512 | >512 | 86 | 84 | 90 | 78 | 84 |
| **Trimethoprim- sulfadiazine** | ≤1..>8 | 8 | >8 | 82 | 79 | 90 | 67 | 84 |

All the isolates were resistant to amoxicillin, amoxicillin-clavulanate, ticarcillin, ticarcillin-clavulanate, temocillin, piperacillin, and piperacillin-tazobactam. *The resistant category also encompasses isolates with intermediate resistance. MIC50: MICs required to inhibit the growth of 50% of organisms. MIC90: MICs required to inhibit the growth of 90% of organisms. ^!^According to CLSI guidelines, *E. coli* isolates harboring OXA-48-like enzymes that test susceptible to meropenem-vaborbactam should still be reported as resistant due to potential in vivo inefficacy.

**S10 Table**. Prediction of human pathogenicity and characterization of virulence genes in carbapenemase-producing *Escherichia coli* from community and healthcare settings.

| **Isolate ID** | **ST** | **Virulence factors** | **Virulence genes (n)** | **Human pathogenicity (%)** |
| --- | --- | --- | --- | --- |
| O101F6 | 617 | *aslA, aamR, anr, csgA, fdeC, fyuA, hha, hlyE, hra, irp2, iss, iucC, iutA, nlpI, sitA, terC, traJ, yehA, yehB, yehC, yehD* | 21 | 97.06 |
| O101F7 | 1284 | *terC, nlpI, fdeC, aslA, capU, iss, hlyE, traT, traJ, anr, csgA, gad, sitA, yehB, yehA, yehC, yehD, astA, iutA, iucC* | 20 | 97.68 |
| O101G5 | 167 | *yghJ, terC, csgA, yehB, yehA, yehC, yehD, fdeC, nlpI, aslA, irp2, fyuA, hha, fimH, gad, traT, traJ, anr, hlyE* | 19 | 97.34 |
| O101G7 | 5842 | *fimH, espY2, kpsE, kpsMII_K5, yehB, yehC, yehD, air, terC, traT, traJ, nlpI, lpfA, csgA, capU, irp2, fyuA, chuA, fdeC, ompT, yfcV, aslA, eilA, hlyE, iss* | 25 | 97.57 |
| O101G8 | 5842 | *hlyE, eilA, traT, kpsE, kpsMII_K5, fimH, irp2, capU, yehB, fdeC, yehD, chuA, terC, espY2, air, yfcV, nlpI, traJ, iss, lpfA, fyuA, ompT, aslA, csgA, hra, aamR, yehA* | 27 | 95.36 |
| O101H1 | 5842 | *fimH, csgA, chuA, aslA, capU, terC, irp2, fyuA, espY2, eilA, air, yfcV, nlpI, yehB, yehC, yehD, lpfA, kpsE, kpsMII_K5, fdeC, traT, traJ, hlyE, ompT, iss* | 25 | 97.88 |
| O101H2 | 617 | *terC, hlyE, iss, nlpI, irp2, fyuA, csgA, aslA, gad, hra, aamR, yehB, yehA, yehC, yehD, fdeC, anr, hha, traT* | 19 | 97.35 |
| O101H4 | 410 | *terC, csgA, yehB, yehA, yehC, yehD, lpfA, irp2, fyuA, fimH, fdeC, nlpI, hha, hlyE, anr* | 15 | 96.97 |
| O101H7 | 361 | *yghJ, irp2, fyuA, terC, fimH, hlyE, yehB, yehA, csgA, fdeC, nlpI, cib, hlyF, ompT, traT, traJ, anr, sitA, hha, capU, iss, iutA, iucC* | 23 | 97.12 |
| O101H9 | 361 | *hlyE, yghJ, csgA, terC, traT, traJ, anr, fimH, nlpI, yehB, yehA, fdeC, irp2, fyuA, capU, iutA, iucC, cib, hlyF, ompT, hha, sitA, iss* | 23 | 97.63 |
| O101I10 | 648 | *fimH, yghJ, hlyE, hra, traT, traJ, terC, gad, csgA, nlpI, capU, fdeC, sitA, iutA, iucC, yehB, yehA, yehC, anr, afaA* | 20 | 97.63 |
| O101J2 | 361 | *terC, irp2, fyuA, nlpI, hlyE, yehB, yehA, yehC, yehD, aslA, hra, aamR, gad, yghJ, csgA, hha, fdeC, capU* | 18 | 97.17 |
| O101J6 | 167 | *yehB, yehA, yehC, yehD, yghJ, hra, aamR, gad, irp2, fyuA, aslA, terC, fdeC, nlpI, hha, csgA, hlyE, capU* | 18 | 97.71 |
| O101H10 | 167 | *eilA, yfcV, kpsE, hlyE, csgA, chuA, yehB, yehC, yehD, fdeC, air, nlpI, terC, aslA, traT, irp2, fyuA, lpfA, afaD, afaC, afaB, afaE, afaA, cib* | 24 | 97.19 |
| O102A6 | 648 | *nlpI, yghJ, terC, capU, hlyE, hra, fimH, fdeC, yehB, yehA, yehC, sitA, csgA, traT, anr, traJ, gad* | 17 | 97.9 |
| O102A4 | 361 | *hra, aamR, yfcV, aslA, chuA, terC, csgA, yehB, yehC, yehD, eilA, fdeC, hlyE, air, lpfA, nlpI, hha, ompT, sitA, traT, anr, iutA, iucC* | 23 | 97.71 |
| O102C1 | 410 | *fimH, csgA, terC, irp2, fyuA, yehB, yehA, yehC, yehD, nlpI, fdeC, lpfA, hlyE, anr, hha* | 15 | 97.19 |
| O102C4 | 10 | *yghJ, fimH, aslA, terC, nlpI, yehB, yehA, yehC, cib, fdeC, gad, iss, hlyE, csgA, shiA, hha, irp2, fyuA* | 18 | 97.17 |
| O102D9 | 940 | *fdeC, lpfA, gad, yghJ, terC, nlpI, irp2, fyuA, hlyE, csgA, capU, yehB, yehA, yehC, yehD, hha* | 16 | 98.11 |
| O90C8 | 167 | *terC, yehB, yehC, yehD, aslA, nlpI, yghJ, irp2, hha, tia, gad, sitA, fdeC, anr, iss, iutA, iucC, csgA, traT, fyuA, papC, papA_F11, hlyE, capU* | 24 | 97.79 |
| O90E9 | 167 | *terC, irp2, fyuA, nlpI, traT, hra, gad, hha, yghJ, csgA, fdeC, aslA, yehB, yehC, yehD, hlyE, iss, aamR, capU, iutA, iucC* | 21 | 97.8 |
| O100H3 | 167 | *yehB, yehC, yehD, terC, hlyE, hra, aamR, yghJ, gad, aslA, fdeC, colE8, nlpI, capU, csgA, hha, iss* | 17 | 97.61 |
| O101A10 | 167 | *yehB, yehC, yehD, aslA, yghJ, gad, tia, terC, csgA, hlyE, nlpI, irp2, fyuA, sitA, fdeC, hha, capU, traT, traJ, iutA, iucC, papC, anr, iss* | 24 | 98.21 |
| O100J3 | 167 | *traT, traJ, anr, terC, aslA, gad, fdeC, yghJ, nlpI, yehB, yehC, yehD, irp2, fyuA, hha, csgA, hlyE, iutA, iucC, sitA, capU, iss, papC, tia, papA_F11* | 25 | 97.64 |
| O101B2 | 167 | *aslA, gad, yehB, yehC, yehD, terC, iss, hra, capU, fdeC, traT, traJ, nlpI, yghJ, hlyE, csgA, aamR, anr, iutA, iucC, hha* | 21 | 97.75 |
| O101D1 | 167 | *yghJ, irp2, fyuA, yehB, yehA, yehC, yehD, gad, hlyE, nlpI, terC, fdeC, hra, aamR, aslA, csgA, capU, hha* | 18 | 97.99 |
| O100G5 | 617 | *terC, nlpI, hra, aamR, irp2, fyuA, yehB, yehA, yehC, yehD, hlyE, iss, aslA, anr, hha, fdeC, csgA, gad* | 18 | 97.45 |
| O101B3 | 617 | *terC, nlpI, fdeC, aslA, gad, hra, iss, yehA, hlyE, irp2, fyuA, csgA, hha, traT* | 14 | 97.77 |
| O101C2 | 617 | *terC, nlpI, csgA, aslA, gad, yehB, yehA, yehC, yehD, hra, aamR, fdeC, irp2, fyuA, hlyE, hha, anr* | 17 | 97.57 |
| O101D3 | 648 | *hlyE, csgA, air, eilA, lpfA, yehB, yehC, yehD, fdeC, terC, nlpI, yfcV, hha, aslA, chuA* | 15 | 97.67 |
| O89J5 | 648 | *terC, yfcV, lpfA, anr, aslA, csgA, chuA, nlpI, eilA, yehB, yehC, yehD, fdeC, hha, gad, air, traT, ompT, hlyE, traJ* | 20 | 97.89 |
| O90A8 | 648 | *nlpI, eilA, traT, hha, terC, yfcV, air, yehC, yehD, aslA, fdeC, lpfA, ompT, hlyE* | 14 | 97.46 |
| O89J6 | 648 | *yfcV, terC, hlyE, csgA, yehB, yehC, yehD, air, eilA, lpfA, fdeC, chuA, aslA, traT, traJ, anr, nlpI, ompT, hha* | 19 | 98.01 |
| O89I2 | 648 | *air, eilA, lpfA, terC, nlpI, yehB, yehC, yehD, yfcV, traT, traJ, chuA, aslA, hlyE, anr, csgA, fdeC, hha, ompT* | 19 | 97.55 |
| O91C3 | 361 | *fimH, hlyE, yghJ, sitA, terC, hra, nlpI, fdeC, csgA, capU, yehB, yehA, yehC, traT, traJ, anr* | 16 | 97.57 |
| O100G9 | 361 | *hra, yehB, yehA, yehC, terC, yghJ, hlyE, traT, traJ, csgA, anr, fimH, sitA, capU, nlpI, fdeC* | 16 | 98.3 |
| O100I10 | 361 | *fimH, hlyE, yghJ, sitA, terC, hra, nlpI, fdeC, csgA, capU, yehB, yehA, yehC, traT, traJ, anr* | 16 | 97.57 |
| O90D7 | 361 | *terC, fimH, traT, gad, nlpI, sitA, fdeC, yehB, yehA, yehC, csgA, capU, hlyE* | 13 | 97.55 |
| O91A5 | 361 | *hra, csgA, fdeC, nlpI, yghJ, aamR, fimH, capU, terC, yehB, yehA, yehC, hlyE, gad, sitA, traT* | 16 | 97.34 |
| O90J2 | 361 | *fimH, terC, fdeC, hlyE, hra, yehB, yehA, yehC, nlpI, capU, aamR, gad, sitA, csgA, anr* | 15 | 97.62 |
| O90A6 | 410 | *terC, lpfA, csgA, hra, gad, yehA, nlpI, fdeC, yehC, yehD, hha, hlyE, sitA, iutA, traJ* | 15 | 96.91 |
| O100F5 | 410 | *terC, hlyE, yehB, yehA, yehC, yehD, csgA, fdeC, fimH, gad, irp2, fyuA, nlpI, lpfA, sitA, iutA, iucC, hha, iss* | 19 | 96.79 |
| O91D1 | 410 | *terC, csgA, fimH, lpfA, fdeC, nlpI, yehB, yehA, yehC, yehD, hlyE, irp2, fyuA, iutA, iucC, cia, hha, iss* | 18 | 96.81 |
| O91D4 | 410 | *terC, fdeC, irp2, lpfA, nlpI, hlyE, yehB, yehA, yehC, yehD, csgA, fimH, hha, gad, sitA, iss, fyuA, cia* | 18 | 97.58 |
| O101E1 | 410 | *yehB, yehA, yehC, yehD, gad, nlpI, terC, hlyE, csgA, anr, lpfA, fimH, fdeC, hha* | 14 | 97.53 |
| O101E5 | 410 | *lpfA, yehB, neuC, yehA, yehC, yehD, terC, fimH, afaC, afaB, afaE, afaD, afaA, gad, csgA, hlyE, irp2, fyuA, nlpI, fdeC, hha, anr, sitA, iutA, iucC* | 25 | 97.64 |
| O101I7 | 1284 | *terC, gad, traT, anr, iss, yehB, yehA, yehC, yehD, nlpI, aslA, fdeC, hra, irp2, fyuA, hha, hlyE, iutA, iucC, sitA, csgA, traJ, capU* | 23 | 97.64 |
| O100J9 | 1284 | *fdeC, terC, nlpI, traT, anr, gad, yehB, yehA, yehC, yehD, aslA, hra, hlyE, irp2, fyuA, hha, traJ, iutA, iucC, sitA, csgA, iss, capU* | 23 | 97.09 |
| O101C7 | 1284 | *gad, yehB, yehA, yehC, yehD, terC, nlpI, fdeC, irp2, fyuA, hha, iss, hra, traJ, aslA, hlyE, csgA, capU, iutA, iucC, sitA, traT, anr* | 23 | 97.57 |
| O101A2 | 617 | *gad, iss, hra, aamR, nlpI, aslA, csgA, terC, hlyE, fdeC, irp2, fyuA, yehB, yehA, yehC, yehD, anr, hha* | 18 | 97.56 |

Using PathogenFinder v2.0.5 (http://genepi.food.dtu.dk/pathogenfinder), *Escherichia coli* isolates were predicted to be a human pathogen.

**S11 Table**. Distribution of the virulence gene *fyuA* in carbapenemase-producing *Escherichia coli* from community and healthcare settings.

| **Isolate ID** | **ST** | ***fyuA* gene** |
| --- | --- | --- |
| O102C4 | 10 | Presence |
| O100J3 | 167 | Presence |
| O101A10 | 167 | Presence |
| O101D1 | 167 | Presence |
| O101G5 | 167 | Presence |
| O101J2 | 167 | Presence |
| O101J6 | 167 | Presence |
| O90C8 | 167 | Presence |
| O90E9 | 167 | Presence |
| O100H3 | 167 | Absence |
| O101B2 | 167 | Absence |
| O101H7 | 361 | Presence |
| O101H9 | 361 | Presence |
| O100G9 | 361 | Absence |
| O100I10 | 361 | Absence |
| O101I10 | 361 | Absence |
| O102A6 | 361 | Absence |
| O90D7 | 361 | Absence |
| O90J2 | 361 | Absence |
| O91A5 | 361 | Absence |
| O91C3 | 361 | Absence |
| O101A2 | 410 | Presence |
| O101E5 | 410 | Presence |
| O101H4 | 410 | Presence |
| O102C1 | 410 | Presence |
| O91D1 | 410 | Presence |
| O91D4 | 410 | Presence |
| O101E1 | 410 | Absence |
| O90A6 | 410 | Absence |
| O100G5 | 617 | Presence |
| O101A2 | 617 | Presence |
| O101B3 | 617 | Presence |
| O101C2 | 617 | Presence |
| O101H2 | 617 | Presence |
| O101F6 | 617 | Presence |
| O101H10 | 648 | Presence |
| O101D3 | 648 | Absence |
| O102A4 | 648 | Absence |
| O89I2 | 648 | Absence |
| O89J5 | 648 | Absence |
| O89J6 | 648 | Absence |
| O90A8 | 648 | Absence |
| O100J9 | 1284 | Presence |
| O101C7 | 1284 | Presence |
| O101I7 | 1284 | Presence |
| O101F7 | 1284 | Absence |
| O101G7 | 5842 | Presence |
| O101G8 | 5842 | Presence |
| O101H1 | 5842 | Presence |
| O102D9 | 940 | Presence |
